# TCP1 expression alters the ferroptosis sensitivity of diffuse large B-cell lymphoma subtypes by stabilising ACSL4 and influences patient prognosis

**DOI:** 10.1038/s41419-024-07001-0

**Published:** 2024-08-22

**Authors:** Shuxia Zhang, Jin Wang, Guanxiang Huang, Xueting Xiao, Shujuan Xu, Ping Weng, Yiting Wang, Huiyun Tian, Huifang Huang, Yuanzhong Chen

**Affiliations:** 1 Department of Hematology, Fujian Institute of Hematology, Fujian Provincial Key Laboratory on Hematology, Fujian Medical University Union Hospital, Fuzhou, 350001 Fujian China; 2 Central Laboratory, Fujian Medical University Union Hospital, Fuzhou, 350001 Fujian China; 3 Laboratory of Gynecologic Oncology, Fujian Maternity and Child Health Hospital, College of Clinical Medicine for Obstetrics & Gynecology and Pediatrics, Fujian Medical University, Fuzhou, 350001 Fujian China; 4 Department of Blood Transfusion, Affiliated Hospital of Putian University, Putian, 351100 Fujian China

**Keywords:** Haematological cancer, Haematological cancer

## Abstract

Diffuse large B-cell lymphoma (DLBCL), an invasive lymphoma with substantial heterogeneity, can be mainly categorised into germinal centre B-cell-like (GCB) and non-GCB subtypes. DLBCL cells are highly susceptible to ferroptosis, which offers an effective avenue for treating recurrent and refractory DLBCL. Moreover, various heat shock proteins are involved in regulating the sensitivity of tumour cells to ferroptosis. Among these proteins, tailless complex polypeptide 1 (TCP1), a subunit of chaperonin-containing T-complex protein-1 (CCT), plays a role in tumour proliferation and survival. Therefore, we explored the role of TCP1 in different DLBCL subtypes, the sensitivity of GCB and non-GCB subtypes to the ferroptosis inducer RAS-selective lethal small molecule 3 (RSL3), and the underlying molecular mechanism. In GCB cells, TCP1 promoted RSL3-induced ferroptosis. Notably, TCP1 could bind with acyl-CoA synthetase long-chain family member 4 (ACSL4), a key enzyme regulating lipid composition and facilitating ferroptosis, to reduce its ubiquitination and degradation. This interaction activated the ACSL4/LPCAT3 signalling pathway and promoted ferroptosis in the GCB subtype. However, in the non-GCB subtype, TCP1 did not act as a positive regulator but served as a predictor of an unfavourable prognosis in patients with non-GCB. In conclusion, our results suggest that in DLBCL, high TCP1 expression enhances the sensitivity of GCB tumour cells to ferroptosis and serves as a marker of poor prognosis in patients with non-GCB DLBCL.

## Introduction

Diffuse large B-cell lymphoma (DLBCL) is the most common type of non-Hodgkin’s lymphoma, and its extensive molecular heterogeneity has hindered the development of targeted therapy [1, 2]. Based on immunohistochemistry (IHC) characteristics, the Hans algorithm is used to classify DLBCL into two subtypes, germinal centre B-cell-like (GCB) and non-GCB, according to CD10, BCL6 and MUM1 expression [3]. Compared with the GCB subtype, the non-GCB subtype exhibits significantly lower 5-year overall survival (OS; 78% vs. 54%) and progression-free survival (76% vs. 48%) [4]. Notably, different DLBCL subtypes are characterised by specific gene expression profiles and mutation patterns. Thus, precision treatment targeting specific molecules and signalling pathways has been recognised as a promising research aim.

The resistance of lymphoma cells to apoptosis is a major reason for combination chemotherapy failures [5]. However, DLBCL cells are highly susceptible to ferroptosis [6, 7]. Hence, patients with DLBCL resistant to apoptosis or refractory to conventional chemotherapy can be treated through ferroptosis induction [8, 9]. Owing to its high lipoxygenase-5 (LOX5) expression, the GCB subtype is especially sensitive to ferroptosis induced by low-dose dimethyl fumarate. Conversely, the activated B-cell-like (ABC) subtype is not sensitive to ferroptosis induced by dimethyl fumarate or RAS-selective lethal small molecule 3 (RSL3), owing to constitutive nuclear factor κB (NF-κB) activation [6]. Therefore, ferroptosis induction enables DLBCL subtype-specific precision treatment.

Molecular chaperones, a class of evolutionarily conserved cytoplasmic proteins, can recognise and bind with partially folded or assembled proteins to facilitate protein folding and transport or prevent protein aggregation, without being involved in the formation of the final product. Thus, molecular chaperones are suitable candidates for novel drug targets and tumour biomarkers. Various heat shock proteins (HSPs) play distinct roles in promoting or inhibiting ferroptosis in diseases [10]. Members of the HSP90 and HSP40 families can promote ferroptosis by enhancing lipid peroxidation [11]. Members of the HSP70 family can inhibit the production of lipid reactive oxygen species (ROS) by promoting the expression of glutathione peroxidase 4 (GPX4) and its antioxidant activity, thereby enhancing cellular resistance to ferroptosis [10]. T-complex protein-1 ring complex (TRiC), also known as chaperonin-containing T-complex protein-1 (CCT), is a member of the chaperone family and comprises eight subunits. CCT1, also known as tailless complex polypeptide 1 (TCP1), belongs to the CCT α subunit and plays a role in tumour proliferation and survival [12, 13]. Notably, TCP1 expression, which is increased in various tumours including breast, oesophageal, and liver cancer and acute myeloid leukaemia, is associated with poor prognosis [14–17]. TCP1 can promote tumour cell proliferation via PI3K/AKT/mTOR pathway activation [18]. In liver cancer, TCP1 regulates the Wnt7b/β-catenin pathway via P53, affecting hepatocyte proliferation and migration [19]. High TCP1 expression is also associated with chemotherapy resistance in ovarian cancer and acute myeloid leukaemia [16, 17].

The present study aimed to investigate the relationship between TCP1 expression and prognosis in different DLBCL subtypes. Moreover, we aimed to delineate the differences in sensitivity to RSL3-induced ferroptosis and explore the role of TCP1 in different subtypes of DLBCL, providing a new perspective and target for the diagnosis and treatment.

## Materials and methods

### Patient samples and follow-up

In this retrospective study, 67 specimens of formalin-fixed paraffin-embedded DLBCL lymph node tissues and 20 specimens of lymph node tissues with reactive hyperplasia were obtained from the Fujian Medical University Union Hospital (Fujian, China) between January 2015 and December 2018 for IHC testing. Patients were followed up once every six months from the time of diagnosis until December 2023. Clinical data were retrieved from detailed electronic medical records. Survival data were based on telephone interviews and the Social Security Death Index. This study was approved by the Ethics Committee of the Fujian Medical University Union Hospital (2023KY155). Informed consent was obtained in writing from all enroled patients.

### Bioinformatics data

Clinical data and gene expression profiles of patients with DLBCL were obtained from the National Centre for Biotechnology Information (NCBI) Gene Expression Omnibus (GEO) (https://www.ncbi.nlm.nih.gov/geo/). Data series were downloaded as normalised expression matrix files and used directly for analysis. A training cohort (GSE53786 dataset, 119 patients) and a validation cohort (GSE10846 dataset, 414 patients) were generated. The GEPIA (http://gepia.cancer-pku.cn/) database was used to explore the relationship between *TCP1* expression and OS; median *TCP1* expression was used as the cut-off for group classification.

### Immunohistochemistry

IHC was performed using rabbit anti-human TCP1 monoclonal antibody (1:100; ab92587, Abcam, Cambridge, MA, USA) to evaluate TCP1 expression in DLBCL lymph node tissues. Average scoring of IHC staining results was performed to account for staining intensity and the proportion of clearly positive tumour cells. Each section was independently scored by two pathologists according to staining intensity and extent as 0, 1, 2, or 3; scores ≤1 and ≥2 were considered low and high TCP1 expression, respectively.

### Cell culture and transfection

The DLBCL cell lines SU-DHL-2 and DB were obtained from the American Type Culture Collection (Manassas, VA, USA). SU-DHL-4 was purchased from the Chinese Academy of Sciences Cell Bank (Shanghai, China). The cells were maintained in RPMI-1640 culture medium (HyClone, Logan, UT, USA) supplemented with 10% foetal bovine serum (Gibco, Thermo Fisher Scientific, Waltham, MA USA). *TCP1* overexpression lentiviral GV341 vector (Ubi-MCS-SV40-blasticidin), *TCP1* short hairpin RNA (shRNA) lentiviral GV112 vector (hU6-MCS-CMV-Puromycin), *ACSL4* shRNA lentiviral GV112 vector (hU6-MCS-CMV-Puromycin), and control lentiviral vectors were constructed by GeneChem (Shanghai, China). The shRNA sequences used were as follows (5′-3′): *TCP1*-shRNA: GGTGTACAGGTGGTTCATTA; *ACSL4*-shRNA: CCAGTGTTGAACTTCTGGAAA.

### RNA isolation and quantitative reverse transcription polymerase chain reaction (qRT-PCR)

Total RNA was extracted from different cells using TRIzol reagent (Invitrogen, Carlsbad, CA, USA), followed by application of the RevertAid First Strand cDNA Synthesis Kit (Thermo Fisher Scientific). qPCR was then performed on the Applied Biosystems 7500/7500 Fast Real-time PCR System (Applied Biosystems, Thermo Fisher Scientific) using the SYBR® Green PCR Master Mix (Roche, Basel, Switzerland). *GAPDH* was used as the internal control for qRT-PCR. Supplementary Table S1 illustrates the primer sequences. The relative expression levels of indicator genes were evaluated using the 2^−ΔΔCt^ method.

### Western blot analysis

Cell lysates were extracted on ice using a radio-immunoprecipitation assay buffer containing 1% phenylmethylsulfonyl fluoride (Beyotime Institute of Biotechnology, Shanghai, China). Protein concentration was measured using the Bicinchoninic Acid Protein Assay Kit (P0011, Beyotime). Equal amounts of protein samples were separated using SDS polyacrylamide gel electrophoresis and transferred to PVDF membranes (Millipore, Burlington, MA, USA). After blocking with 5% non-fat dry milk in Tris-buffered saline with 0.1% Tween 20 for 1 h, the membranes were incubated with antibodies overnight at 4 °C, followed by incubation with secondary antibodies for 1 h at room temperature. Protein bands were then observed using enhanced chemiluminescence (Bridgen, Beijing, China), and the blots were visualised using the ChemiDoc Touch Imaging System (Bio-Rad Laboratories, Hercules, CA, USA). TCP1-alpha (ab92587), ACSL4 (ab155282), LPCAT3 (ab232958), GAPDH (ab181602) all purchased from Abcam (Cambridge, MA, USA), and ubiquitin (P4D1, mouse monoclonal; #3936; Cell Signalling Technology, Danvers, MA, USA) antibodies were used.

### Cytotoxicity and cell death assays

DLBCL cells (1 × 10^4^ cells/well) were plated into 96-well plates. After treatment with the RSL3 (2.5 μM in DB cells and 0.4 μM in SU-DHL-4 cells), 10 μL of the Cell Counting Kit-8 (CCK8; DOJINDO, Shanghai, China) solution was added to each well at the specified times and incubated for 1 h at 37 °C. Relative optical density was then measured at 450 nm using a Model 680 Microplate Reader® (Bio-Rad Laboratories). Some cells were treated with 10 μM ferrostatin-1 (FS-1), 1 μM ZVAD-FMK, 1 μM necrosulfonamide, or 10 μM rosiglitazone (ROSI; ACSL4 inhibitor) for 1 h, followed by (1S, 3R)-RSL3 (all Selleckchem, Houston, TX, USA) treatment for 48 h and a CCK8 test.

### Intracellular ROS assay

Intracellular ROS levels were determined using dichlorodihydrofluorescein diacetate (DCFH-DA; D6883, Sigma-Aldrich, St. Louis, MO, USA). In brief, 1 × 10^5^/ml DLBCL cell lines were maintained in 6-well plates with 5 μM DCFH-DA in a serum-free medium at 37 °C in the dark for 30 min. After rinsing three times with phosphate-buffered saline (PBS), the cells were suspended in 500 μL of PBS and analysed using a flow cytometer (BD Biosciences FACS Celesta, San Jose, CA, USA). The software FlowJo 10.7.2 was used to analyse the results.

### Lipid ROS assay

The lipid ROS levels in cells were assessed using the C11-BODIPY dye (GC40165, GlpBio, CA, USA). Cells were resuspended in a serum-free medium supplemented with 10 μM C11-BODIPY581/591 and incubated at 37 °C for 30 min. The cells were then collected via centrifugation and excess dye was removed by washing thrice with sterile PBS. C11-BODIPY581/591 oxidation resulted in a shift of the fluorescence emission peak from ~590 to ~510 nm. Lipid peroxidation was analysed using a flow cytometer (BD Biosciences FACS Celesta, San Jose, CA, USA); the software FlowJo 10.7.2 was used to analyse the results.

### Malondialdehyde (MDA) assay

MDA concentrations can be used to reflect lipid oxidation levels. The MDA concentration of DLBCL cells was determined using the thiobarbituric acid method. The Cellular MDA Assay Kit (S0131; Beyotime) was used to measure the relative MDA concentration in cells or tumour lysates. MDA was calculated based on cellular protein concentration, and the MDA content in the original sample was expressed as the protein content per unit weight (μM/mg).

### Iron assay

The fluorescence of the Phen Green SK (PGSK) dye is quenched in a quantitatively proportional manner in the presence of iron. Following the removal of the cell culture medium, cells were washed thrice with sterile PBS and treated with 10 μM Phen Green™ SK reagent (cat. no. P14313, Invitrogen, Thermo Fisher Scientific, Inc., Eugene, OR, USA) in serum-free medium at 37 °C for 1 h. Quenching was observed under a confocal laser scanning microscope (Olympus FluoView 1000, Olympus Life Science Europa GmbH, Hamburg, Germany). Fluorescence was determined in five randomly selected fields using the ImageJ software, an open-source Java image processing program from the National Institute of Health.

### Transmission electron microscopy (TEM)

After treatment with RSL3 or dimethylsulfoxide for 24 h, cells were fixed in 4% paraformaldehyde (P0099; Beyotime) for 2 min. The cells were harvested by centrifugation at 1000 × *g*, and 2.5% glutaraldehyde (P1126; Solarbio, Beijing, China) was cautiously added to the cell pellet. Finally, the samples were cut into ultrathin sections, and changes in mitochondrial morphology were observed using TEM (TECNA I20; Philips, Eindhoven, Netherlands).

### Sample preparation for in vitro mass spectrometry-based non-targeted lipidomics

For lipid extraction, 5 × 10^6^ DLBCL cells were homogenised in 250 μL of cold ethanol containing 0.1% butylated hydroxytoluene using a microtip sonicator. The homogenised sample was then transferred to a fresh glass tube containing 850 μL of cold methyl tert-butyl ether and vortexed for 30 s. To increase the extraction efficiency, samples were incubated overnight on a shaker at 4 °C. The following day, 200 μL of cold water was added to each sample and incubated on ice for 20 min, followed by centrifugation at 3000 × *g*, 4°C for 20 min. The organic layer was collected, dried under a gentle stream of nitrogen gas on ice, and stored at –80 °C for liquid chromatography-mass spectrometry (LC-MS/MS) analysis.

### Immunofluorescence staining and confocal fluorescence microscopy

Cells were centrifuged, smeared on glass slides, and fixed with 100% ice-cold methanol for 20 min at –20 °C. After rinsing with PBS, the slides were blocked with blocking buffer (1× PBS/5% normal serum/0.3% Triton X-100) for 1 h. The cells were then incubated with rabbit anti-TCP1 antibody (1:50, ab92587,Abcam) and mouse anti-ACSL4 antibody (1:50, sc365230, Santa Cruz Biotechnology, Dallas, TX, USA) overnight at 4 °C, followed by incubation with goat anti-rabbit IgG (Alexa Fluor 647; ab150083, Abcam) and goat anti-mouse IgG (Alexa Fluor 488;bs-0296G-AF488,Bioss Antibodies, Woburn, MA, USA) secondary antibodies at 25 °C for 1 h. Nuclei were counterstained using 4’,6-diamidino-2-phenylindole (DAPI) for 5 min and blocked with an antifade reagent. Fluorescence staining of ACSL4 and TCP1 in tumours was performed according to the manufacturer’s protocol (Servicebio, Wuhan, China). Finally, image acquisition and analysis were performed using a confocal fluorescence microscope (TCS SP8; Leica, Weltzar, Germany).

### Co-immunoprecipitation (co-IP)

DLBCL cells were lysed in ice-cold IP lysis/wash buffer supplemented with a cocktail of protease and phosphatase inhibitors. Cell lysates were mixed with anti-TCP1 (ab92587, Abcam), anti-ACSL4 (sc365230, Santa Cruz Biotechnology), anti-ubiquitin (#3936; Cell Signalling Technology), or IgG antibody (ab6721 or ab6789, Abcam) in 20 μL of protein A/G plus agarose (#88804; Thermo Fisher Scientific) and incubated overnight on a shaker at 4 °C. Subsequently, 20 μL of protein A/G plus agarose was added, followed by incubation for another 6 h. The IP beads were then rinsed three times with ice-cold lysis buffer and boiled in 50 μL of 1× SDS sample buffer at 95 °C for 8 min. Finally, western blotting was performed to analyse the immunoprecipitated protein complexes.

### Statistical analysis

Statistical analyses were conducted using SPSS 23.0 (SPSS Inc., Chicago, IL, USA USA) and GraphPad Prism 8.0 (GraphPad Software Inc., La Jolla, CA, USA). All data were expressed as mean ± standard deviation (SD), and comparisons were performed using the two-tailed unpaired Student’s *t* test or one-way analysis of variance (ANOVA). Pearson’s chi-squared test was employed to analyse the relationship between TCP1 expression and pathological variables. Survival analysis was carried out using the Cox proportional hazard regression model, Kaplan–Meier method, and the log-rank test. Statistical significance was set at *p* < 0.05.

## Results

### *TCP1* enhances the sensitivity of GCB DLBCL cells to the ferroptosis inducer RSL3

**Fig. 1:**
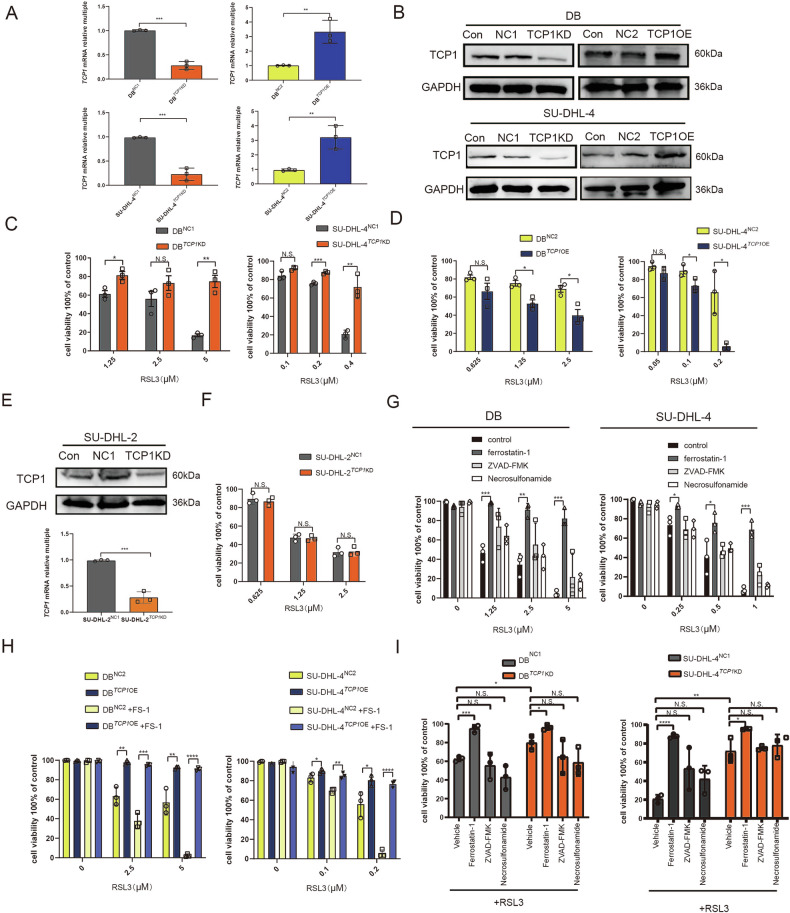
*TCP1* promotes RSL3-induced ferroptosis in GCB DLBCL cells. **A** qPCR analysis of *TCP1* mRNA expression levels in *TCP1*-knockdown and *TCP1*-overexpressing DB and SU-DHL-4 cells. **B** Western blotting of TCP1 protein expression levels in *TCP1*-knockdown and *TCP1*-overexpressing DB and SU-DHL-4 cells. **C** CCK8 assay of cell viability after RSL3 treatment of *TCP1*-knockdown DB and SU-DHL-4 cells. **D** CCK8 assay of cell viability after RSL3 treatment of *TCP1*-overexpressing DB and SU-DHL-4 cells. **E** qPCR and western blot analyses of the effect of *TCP1* knockdown in SU-DHL-2 cells. **F** CCK8 assay of cell viability after RSL3 treatment of *TCP1*-knockdown SU-DHL-2 cells. **G** Effect of the combined treatment with RSL3 and various inhibitors (FS-1, ZVAD-FMK, or necrosulfonamide) on the cell viability of DB and SU-DHL-4 cells. **H** Effect of the combined treatment with RSL3 and various inhibitors on the cell viability of *TCP1*-overexpressing DLBCL cells. **I** Effect of the combined treatment with RSL3 and various inhibitors (FS-1, ZVAD-FMK, or necrosulfonamide) on the cell viability of *TCP1*-knockdown DLBCL cells. Data are presented as mean ± SD (*n* = 3 experiments); *p < 0.05, **p < 0.01, ***p < 0.001.

We selected the GCB cell lines DB and SU-DHL-4 for *TCP1* knockdown and overexpression and confirmed the knockdown and overexpression efficiency were confirmed using qRT-PCR and western blot analyses (Fig. 1A, B). Compared to cells transfected with empty vectors, *TCP1* knockdown inhibited cell death induced by different RSL3 concentrations (Fig. 1C), whereas *TCP1* overexpression enhanced RSL3-induced cell death (Fig. 1D). In the non-GCB cell line SU-DHL-2, *TCP1* knockdown (Fig. 1E) did not affect the sensitivity to RSL3 (Fig. 1F).

Treatment with the ferroptosis inhibitor FS-1 significantly reversed RSL3-induced growth inhibition in DB and SU-DHL-4 cells. However, ZVAD-FMK, a pan-caspase inhibitor, and necrosulfonamide, a potent necroptosis inhibitor targeting mixed lineage kinase domain-like protein, did not significantly reverse RSL3-induced growth inhibition in DB and SU-DHL-4 cells (Fig. 1G). In *TCP1*-overexpressing cells, FS-1 significantly reversed the growth inhibition effect induced by different concentrations of RSL3 (Fig. 1H). FS-1 also reversed RSL3-induced growth inhibition in the absence of TCP1, whereas this effect was not significant with ZVAD-FMK and necrosulfonamide (Fig. 1I).

**Fig. 2:**
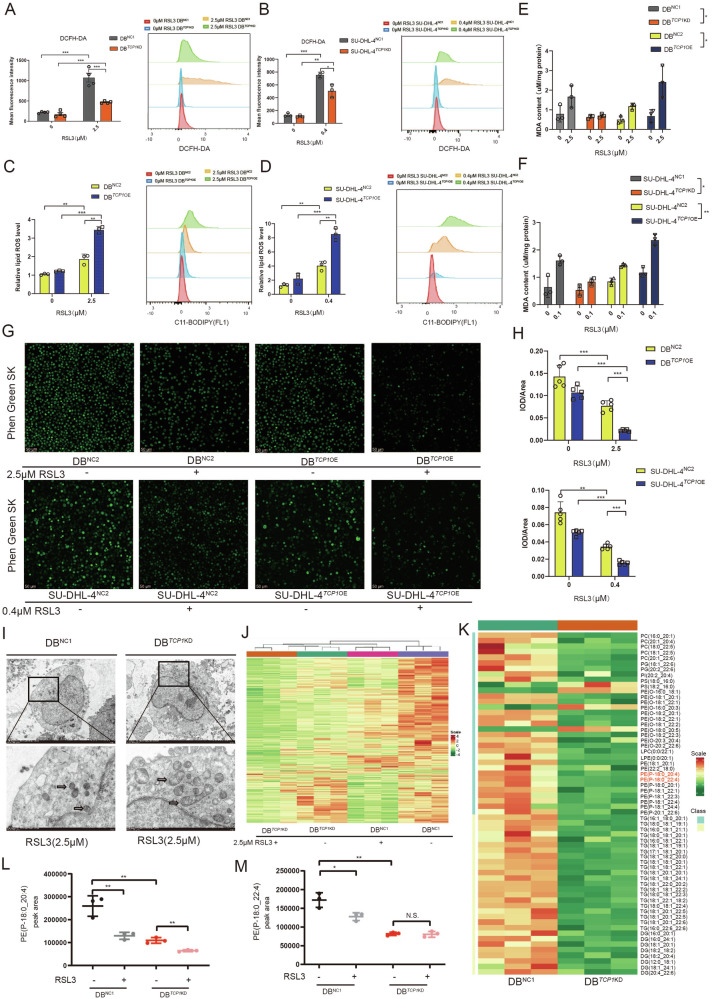
*TCP1* alters the lipid composition of GCB DLBCL. **A**, **B** ROS levels of RSL3-treated *TCP1*-knockdown DB and SU-DHL-4 cells. **C**, **D** Lipid peroxidation levels in RSL3-treated *TCP1*-overexpressing DB and SU-DHL-4 cells measured using C11-BODIPY (581/591) staining. **E**, **F** MDA activity of RSL3-treated *TCP1*-knockdown and *TCP1*-overexpressing DB and SU-DHL-4 cells. **G**, **H** Representative images of Phen Green SK staining in RSL3-treated TCP1-overexpressing DB and SU-DHL-4 cells as observed using confocal laser scanning microscopy (CLSM) (scale bars: 50 μm). **I** TEM images showing the mitochondrial morphology of the control and *TCP1*-knockdown groups following RSL3 treatment (24 h). The control group shows increased mitochondrial membrane density and shrinkage, whereas the *TCP1*-knockdown group shows no significant changes in mitochondrial morphology (black arrows). Scale bar: 2 μm. **J** Heatmap of lipid composition differences in RSL3-treated *TCP1*-knockdown DB cells. **K** Heatmap of lipid composition differences in *TCP1*-knockdown DB cells. **L**, **M** Relative content of PE-18:0/22:4 and PE-18:0/20:4 in RSL3-treated DB cells of the empty vector and *TCP1*-knockdown groups compared with the untreated group. Data are presented as mean ± SD; *p < 0.05, **p < 0.01, ***p < 0.001.

RSL3 also significantly decreased ROS levels in the *TCP1*-knockdown group compared with that in the empty vector group (Fig. 2A, B). Probing with C11-BODIPY showed that *TCP1* overexpression elevated the levels of lipid ROS in RSL3-induced DB and SU-DHL-4 cells (Fig. 2C, D). In *TCP1-deficient* GCB cells, RSL3 decreased MDA levels, whereas the opposite effect was observed in *TCP1*-overexpressing cells (Fig. 2E, F). In addition, analysis of iron adsorption verified that TCP1 promoted RSL3-induced ferroptosis in GCB DLBCL cells. Notably, *TCP1* overexpression markedly decreased the fluorescent intensity of PGSK staining in DB and SU-DHL-4 cells in an RSL3 treatment-dependent manner (Fig. 2G, H). TEM findings indicated that cells in the RSL3-treated empty vector group exhibited mitochondrial membrane rupture and bubbling, volume shrinkage, significantly increased membrane density, reduced or absent mitochondrial cristae, and outer mitochondrial membrane rupture. Compared with the empty vector group, cells in the RSL-treated *TCP1*-knockdown group exhibited a more intact mitochondrial structure, a larger size, and more cristae (Fig. 2I). These results indicate that *TCP1* promotes RSL3-induced ferroptosis in the GCB subtype of DLBCL.

### *TCP1* alters the lipid composition of GCB DLBCL cells

We determined the changes in lipid composition following *TCP1* knockdown to examine the causes underlying the effects of *TCP1* on ferroptosis sensitivity. In LC-MS/MS experiments, RSL3-treated DLBCL cells exhibited significantly reduced total lipid levels (Fig. 2J). Compared with empty vector transfection, *TCP1* knockdown significantly decreased the levels of 58 lipid types (Fig. 2K), especially phosphatidylethanolamine (PE) containing arachidonoyl (AA; C 20:4) and adrenoyl (Ada; C 22:4) moieties, which may play a direct role in driving ferroptosis. This result suggests that *TCP1* knockdown reduces PE-18:0/22:4 and PE-18:0/20:4 levels, thereby decreasing cell sensitivity to RSL3-induced ferroptosis (Fig. 2L, M).

### *TCP1* upregulates ACSL4 protein levels for enhanced RSL3 sensitivity

**Fig. 3:**
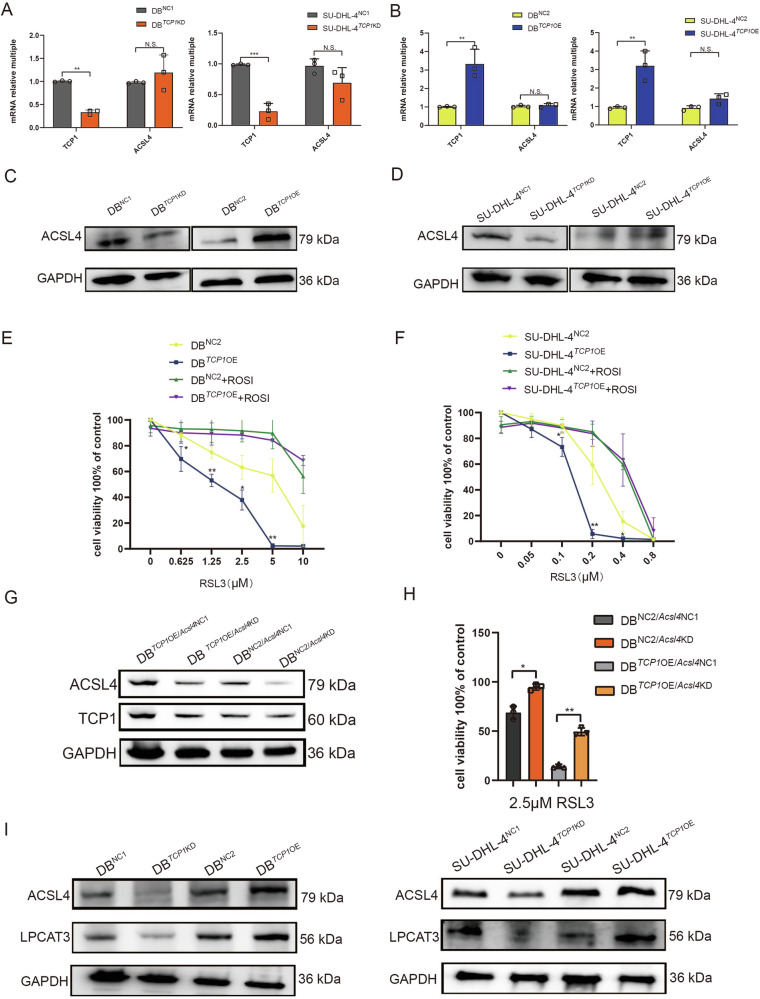
*TCP1* regulates ferroptosis via the regulation of changes in ACSL4 protein level. **A**, **B** qPCR analysis of *ACSL4* mRNA expression levels in *TCP1*-knockdown and *TCP1*-overexpressing DB and SU-DHL-4 cells. **C**, **D** Western blotting of ACSL4 protein expression levels in *TCP1*-knockdown and *TCP1*-overexpressing DB or SU-DHL-4 cells. **E**, **F** Effect of the treatment with RSL3 alone or combined with ROSI on the cell viability of *TCP1*-overexpressing DB and SU-DHL-4 cells. **G** Western blot verifying the successful *ACSL4* knockdown in *TCP1*-overexpressing DB cells. **H** Effect of RSL3 treatment on the cell viability of *ACSL4*-knockdown *TCP1*-overexpressing DB cells. **I** Western blot analysis of the protein expression levels of ACSL4 and LPCAT3 in the different groups. Data are presented as mean ± SD (*n* = 3 experimental groups); *p < 0.05, **p < 0.01, ***p < 0.001.

ACSL4 can promote the synthesis of AA- and AdA-CoA from AA and Ada, ultimately generating the lipid peroxide (LPO) PE-AA/ADA-OOH and causing ferroptosis. In the present study, qRT-PCR revealed no significant changes in *ACSL4* expression following *TCP1* knockdown or overexpression (Fig. 3A, B). However, western blotting indicated that ACSL4 expression was downregulated after *TCP1* knockdown and upregulated after *TCP1* overexpression (Fig. 3C, D). Moreover, we administered ROSI, which inhibits ACSL4 expression, to observe whether *TCP1* regulates RSL3-induced ferroptosis in DLBCL cells via ACSL4. Compared to control, *TCP1* overexpression increased the sensitivity of DB and SU-DHL-4 cells to RSL3-induced ferroptosis. When combined with ROSI, *TCP1*-overexpressing groups exhibited significantly enhanced resistance to RSL3-induced ferroptosis (Fig. 3E, F).

We subjected DB cells to *TCP1* overexpression, followed by *ACSL4* knockdown (Fig. 3G). After treatment with 2.5 μM RSL3, both *ACSL4* knockdown groups (DB^NC2^/^*Acsl4*KD^ and DB^*TCP1*OE^/^*Acsl4*KD^) became resistant to RSL3-induced cell death (Fig. 3H). The upregulation of the ACSL4/LPCAT3 signalling pathway promotes ferroptosis [20–22]. Therefore, we determined using western blotting the effects of *TCP1* knockdown and overexpression on ACSL4 and LPCAT3 expression. *TCP1* knockdown significantly downregulated ACSL4 and LPCAT3 expression levels, whereas *TCP1* overexpression had the opposite effect (Fig. 3I).

**Fig. 4:**
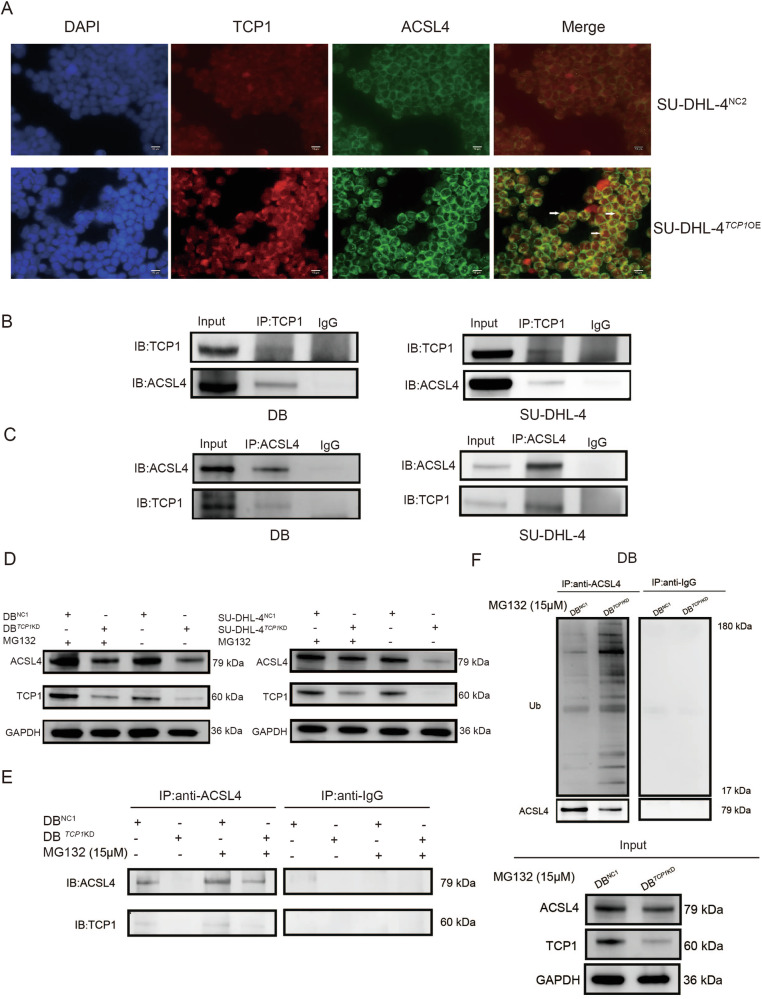
TCP1 binds with and stabilises ACSL4 protein to reduce ACSL4 ubiquitination and degradation. **A** Fluorescence microscopic observation of the co-localisation of TCP1 (red) and ACSL4 (green) in *TCP1*-overexpressing SU-DHL-4 cells. Nuclei were counterstained with DAPI (blue). **B**, **C** Verification of the interaction between ACSL4 and TCP1 through co-IP in DB and SU-DHL-4 cells. **D** Western blot analysis of ACSL4 expression levels in *TCP1*-knockdown DB and SU-DHL-4 cells in the presence or absence of the proteasome inhibitor MG132 (20 μM). **E** Detection of TCP1 and ACSL4 expression levels via immunoprecipitation (IP) of proteins from each lysate sample with anti-ACSL4 antibody or control antibody (rabbit IgG) in the presence or absence of the proteasome inhibitor MG132 (20 μM). **F** IP of lysates with anti-ACSL4 antibody or control antibody (rabbit IgG) in the presence of the proteasome inhibitor MG132 (20 μM), and analysis of ubiquitinated ACSL4 in IP complexes using anti-ACSL4 and anti-ubiquitin antibodies. Data are presented as mean ± SD (*n* = 3 experiments); *p < 0.05, **p < 0.01, ***p < 0.001.

To further clarify how *TCP1* upregulates ACSL4, we examined the interactions between TCP1 and ACSL4. Immunofluorescence experiments revealed that TCP1 and ACSL4 co-localised in the cytoplasm (Fig. 4A). Co-IP experiments using DLBCL cells demonstrated that the IP of TCP1 led to the co-IP of ACSL4 (Fig. 4B), and vice versa (Fig. 4C). These findings suggest direct interactions between TCP1 and ACSL4. Moreover, the proteasome inhibitor MG132 (20 μM) significantly suppressed ACSL4 protein downregulation induced by *TCP1* knockdown (Fig. 4D). We also performed IP using anti-ACSL4 antibody or control antibody (rabbit IgG) on equal amounts of whole-cell lysates, followed by western blotting using anti-ACSL4 or anti-TCP1 antibody. Compared with control cells, *TCP1* knockdown reduced ACSL4 expression levels, and this decrease was partially abolished by MG132 (Fig. 4E). Following ACSL4-IP, the precipitate of the *TCP1* knockdown group exhibited a lower non-ubiquitinated ACSL4 content and a higher polyubiquitinated ACSL4 content than those of the control group (Fig. 4F). Conversely, western blotting using anti-ACSL4 or anti-ubiquitin antibodies did not reveal specific bands in the control IgG IP. These findings provide direct evidence that TCP1 stabilises the ACSL4 protein by inhibiting its ubiquitination and degradation.

### TCP1 in the prognostic evaluation of non-GCB and GCB DLBCL

**Fig. 5:**
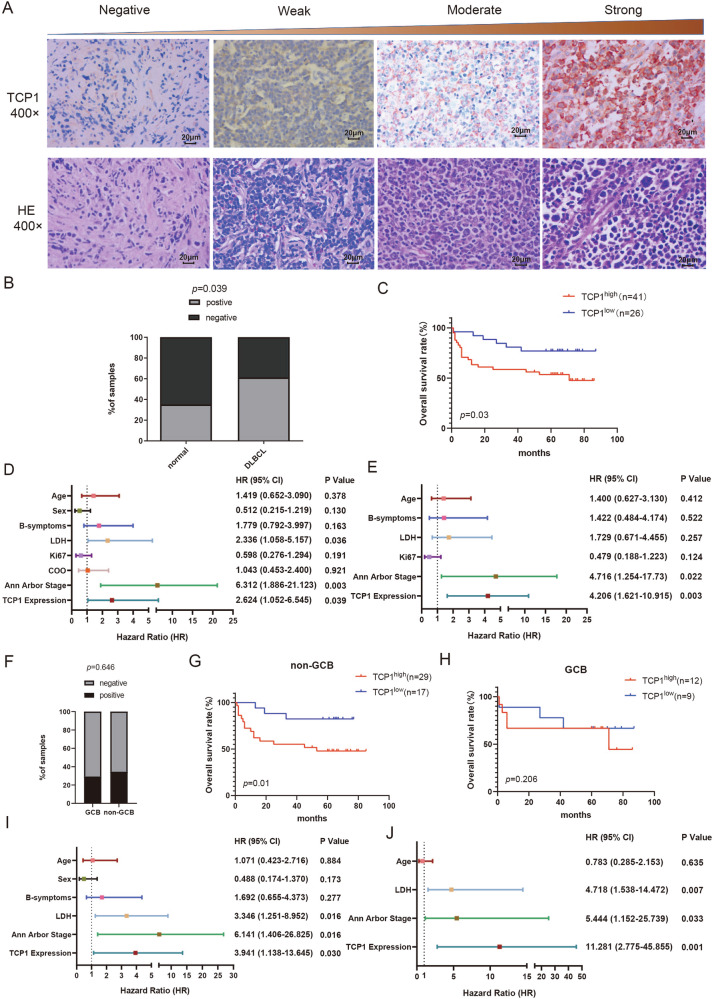
Relationship between TCP1 expression and prognosis in different DLBCL subtypes. **A** Representative images of TCP1 immunohistochemistry (IHC) and H&E staining of lymph node tissues from patients with DLBCL. Scale bar: 20 μm. **B** IHC evaluation of the differential expression of TCP1 protein between DLBCL and reactive hyperplasia samples (DLBCL, *n* = 67; reactive hyperplasia, *n* = 20). **C** Kaplan–Meier survival analysis of TCP1 expression levels in DLBCL lymph node tissues and patient OS. **D**, **E** Forest plot summary of univariate and multivariate analyses on TCP1 expression and other clinical parameters. **F** Differential expression of TCP1 protein levels in the GCB and non-GCB subtypes. **G**, **H** Kaplan–Meier survival analysis of TCP1 expression in DLBCL lymph node tissues and OS of patients with GCB or non-GCB. **I**, **J** Forest plot summary of univariate and multivariate analyses on TCP1 expression and other clinical parameters in the non-GCB subtype. HR hazard ratio, 95% CI 95% confidence interval, OS overall survival. *p < 0.05, **p < 0.01, ***p < 0.001.

To examine TCP1 expression in DLBCL, we performed IHC to detect the TCP1 levels in 67 cases of DLBCL and 20 specimens of lymph node tissues with reactive hyperplasia. The clinical data of the patients are shown in Supplementary Table 2. Representative IHC and HE staining (Fig. 5A) revealed high TCP1 protein expression in 61.2% (41/67) of patients with DLBCL, which was higher than that in lymph node tissues with reactive hyperplasia (35%, 7/20; Fig. 5B). To analyse the clinical significance of TCP1 in DLBCL, we assessed the relationship between TCP1 protein expression and clinicopathological parameters. High TCP1 expression was associated with a higher International Prognostic Index (*p* = 0.033, chi-squared test; Table 1). Kaplan–Meier survival curves also demonstrated that patients with high TCP1 levels had significantly shorter OS than those with low TCP1 levels (log-rank *p* = 0.03, Fig. 5C). Univariate Cox regression analysis indicated that high LDH levels, Ann Arbor Stage III-IV, and high TCP1 expression were significantly associated with an increased risk of mortality, with hazard ratios (HRs) of 2.336 (*p* = 0.036), 6.312 (*p* = 0.003), and 2.624 (*p* = 0.039), respectively (Fig. 5D). Multivariate Cox regression analysis indicated that Ann Arbor Stage III-IV (HR = 4.716, *p* = 0.022) and high TCP1 expression (HR = 4.206, *p* = 0.003) were significantly associated with an increased mortality risk (Fig. 5E).

**Table 1:** Correlation between TCP1 expression and DLBCL clinicopathological characteristics.

	TCP1 expression		
Characteristics	Positive	Negative	*p*-value
Total	41	26	
Sex			
Male	24	16	1
Female	17	10	
Age			
≤60	19	14	0.621
>60	22	12	
Ann Arbor Stage			
I-II	15	9	0.541
III-IV	26	17	
LDH			
Normal	25	11	0.208
>245IU/l	16	15	
Bone marrow involvement			
Yes	3	1	0.956
No	38	25	
B symptoms			
No	31	18	0.584
Yes	10	8	
COO			
GCB	12	9	0.646
non-GCB	29	17	
Ki67(%)			
>70	29	13	0.087
≤70	12	13	
IPI			
Low risk group	11	8	0.033^*a^
Low and medium risk group	12	8	
High and medium risk group	6	9	
High risk group	12	1	

*IPI* international prognostic index; *LDH* lactate dehydrogenase.

*p < 0.05.

^a^Fisher’s exact test.

We further analysed the value of TCP1 expression in the prognostic evaluation of different DLBCL subtypes. TCP1 expression levels were not significantly different between GCB and non-GCB subtypes (Fig. 5F). However, in patients with non-GCB, high TCP1 expression was associated with shorter OS (log-rank *p* = 0.01, Fig. 5G), whereas this association was not significant in patients with GCB (log-rank *p* = 0.206, Fig. 5H). Moreover, in the non-GCB subtype, univariate and multivariate analyses revealed that high LDH levels, Ann Arbor Stage III-IV, and high TCP1 expression were significantly associated with increased risk of mortality (Fig. 5I, J).

**Fig. 6:**
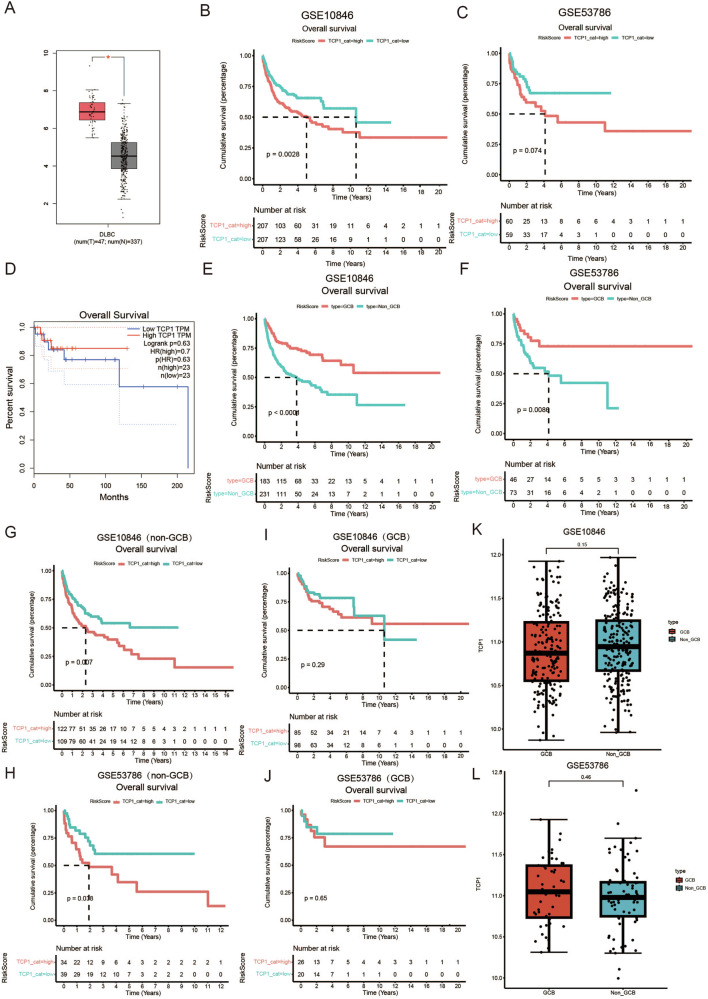
Dataset verification of the relationship between *TCP1* expression and prognosis in different DLBCL subtypes. **A** Differential expression of *TCP1* mRNA between healthy individuals and patients with DLBCL in the GEPIA 2.0 database. **B**, **C** Kaplan–Meier analysis of TCP1 expression and patient OS in the GSE10846 and GSE53786 datasets. **D** Kaplan–Meier analysis of TCP1 expression and patient OS in GEPIA 2.0. **E**, **F** Kaplan–Meier analysis of overall survival (OS) for GCB vs. non-GCB subtypes in the GSE10846 or GSE53786 datasets. **G**, **H** Kaplan–Meier analysis of OS for TCP1 expression in non-GCB in the GSE10846 or GSE53786. **I**, **J** Kaplan–Meier analysis of OS for TCP1 expression in GCB in the GSE10846 or GSE53786. **K**, **L** Differential expression of *TCP1* mRNA levels between the GCB and non-GCB subtypes in the GSE10846 and GSE53786 datasets. *p < 0.05, **p < 0.01, ***p < 0.001.

To further verify the influence of *TCP1* expression on DLBCL, we searched the GEPIA database and found that in The Cancer Genome Atlas (TCGA), *TCP1* expression was significantly elevated in patients with DLBCL (Fig. 6A). The GEO database (GSE10846 and GSE53786) was selected for validation; the clinical characteristics of these patients are shown in Table 2. Contrary to our expectations, high *TCP1* expression was associated with poor prognosis in GSE10846 (Fig. 6B), but not in GSE53786 (Fig. 6C). We then used GEPIA2 to analyse *TCP1* mRNA expression in patients with DLBCL retrieved from the TCGA database, which also revealed that *TCP1* expression was not associated with DLBCL prognosis (Fig. 6D).

**Table 2:** The clinical characteristics of GEO datasets.

Characteristics	GSE53786	GSE10846
Sample size	119	414
Age, years mean (SD)	61.50 (14.59)	61.14 (15.43)
Gender		
Male	67	224
Female	47	172
Unknown	5	18
Stage		
I	16	66
II	35	122
III	31	97
IV	33	121
Unknown	4	8
ECOG PS > 1	33	93
Cell of orign		
GCB	46	183
Non-GCB	73	231
Over survival		
Time, years mean (SD)	3.03 (3.47)	3.17 (3.11)
Death	44	165

*ECOG PS* The Eastem Cooperative Oncology Group performance score.

It is widely accepted that patients with non-GCB have a significantly poorer prognosis compared to those with GCB [4]. Following grouping based on the case information, this viewpoint was confirmed in the GSE10846 and GSE53786 datasets (Fig. 6E, F). In patients with non-GCB, high *TCP1* expression was significantly associated with shorter OS (log-rank *p* = 0.007 and *p* = 0.038, Fig. 6G, H, respectively), whereas in patients with GCB, *TCP1* expression was not related to OS (log-rank *p* = 0.29 and *p* = 0.65, Fig. 6I, J, respectively). Furthermore, our findings demonstrated no significant difference in *TCP1* expression levels between non-GCB and GCB subtypes (Fig. 6K, L). Therefore, TCP1 may fulfil different functions in the two DLBCL subtypes.

## Discussion

Apoptosis resistance and molecular heterogeneity are major challenges in the treatment of DLBCL. Based on molecular heterogeneity, the combination of targeted drugs guided by genetic subtypes with immunotherapy has achieved encouraging clinical responses [23, 24]. In the present study, we found that TCP1 expression had different implications for GCB and non-GCB DLBCL, as it only predicted poor prognosis in non-GCB subtypes. Furthermore, we demonstrated that in GCB DLBCL, TCP1 binds with and stabilises ACSL4 and regulates RSL3-induced ferroptosis. Therefore, studying the function of *TCP1* in GCB/non-GCB subtypes of DLBCL will provide a theoretical basis for the precision treatment of patients with different DLBCL subtypes.

Precision treatment according to DLBCL subtypes can benefit patients. In the non-GCB subtype, mutations in the active B cell receptor pathway can lead to constitutive NF-κB activation, inhibiting apoptosis and promoting uncontrolled cell division, thereby resulting in progressive disease or insensitivity to chemotherapy [5, 25, 26]. Therefore, in recurrent and refractory DLBCL, Burton’s tyrosine kinase inhibitor ibrutinib elicits a significantly higher response rate in the non-GCB subtype than in the GCB subtype [25, 27]. Furthermore, pSTAT3 positivity is associated with a favourable prognosis in patients with GCB [28]. However, pSTAT3 can facilitate tumour dissemination and progression by regulating microtubule dynamics in ABC DLBCL, which, in turn, will promote disease progression of the non-GCB subtype [29, 30]. Positive regulatory domain zinc finger protein 1 (PRDM1) is not differentially expressed between GCB and non-GCB subtypes, but it is frequently inactivated by structural changes in ABC DLBCL, although it exhibits no such effect in GCB or unclassified DLBCL [31, 32]. Homozygous or heterozygous PRDM1 deletion is an unfavourable prognostic factor in non-GCB DLBCL [33]. In the present study, TCP1 was similar to pSTAT3 and PRDM1, in that it fulfilled distinct functions in different DLBCL subtypes. TCP1 forms with seven other subunits a TRiC complex that functions as a chaperone protein [34], and targeting TCP1 may suppress the proliferation and survival of tumour cells. In our study, in GCB cell lines, *TCP1* knockdown decreased the levels of RSL3-induced ROS and MDA, reduced mitochondrial damage, and inhibited ferroptosis, whereas *TCP1* overexpression promoted RSL3-induced DLBCL cell death. Thus, TCP1 acts as a positive regulator of ferroptosis. However, in the non-GCB DLBCL cell line, TCP1 was not involved in regulating ferroptosis. TCP1 may play different functions in different subtypes of DLBCL.

RSL3, the most commonly used ferroptosis inducer [35, 36], directly suppresses GPX4 and induces the accumulation of iron-dependent lethal LPOs, thereby triggering cell death. ACSL4 and GPX4 are two major enzymes that positively and negatively regulate ferroptosis, respectively [37]. In cancer cells, the labile iron pool and increase in lipid content and ACSL4 expression may lead to a GPX4-dependent increase, resulting in vulnerability to GPX4 inhibitors. Regulating ACSL4 expression affects the sensitivity of cell lines to RSL3 [38, 39]. In addition, retinoblastoma (RB1)-intact PCa cells have nearly undetectable levels of ACSL4; thus, a low basal level of lipid peroxidation. Owing to this weak ferroptotic potential, RB1-intact cells do not undergo notable ferroptosis, even when treated with GPX4 inhibitors. Upon RB1 loss, E2F transcription factors were shown to induce ACSL4 expression, with cells becoming sensitive to the GPX4 inhibitor RSL3 [40]. In our study, TCP1 was also confirmed to lead to the accumulation of PUFA-PL on the cell membrane by stabilising the expression of ACSL4; conversely, this strong ferroptotic potential was unblocked when TCP1-overexpressing GCB DLBCL cells were treated with GPX4 inhibitors, resulting in ferroptosis.

Polyunsaturated fatty acids (PUFAs), such as AA and Ada (both ω-6 PUFAs), are the main LPO substrates during ferroptosis, which can cause damage to membrane structure and function [41, 42]. Through lipidomics, we found that *TCP1* knockdown in DB cells downregulated the expression of lipid components, including PE-18:0/22:4 and PE-18:0/20:4. PUFA production and synthesis are regulated by various enzymes, especially ACSL4 and LPCAT3. By incorporating PUFAs, especially PE, into cellular phospholipids, these enzymes can promote the production of lethal LPOs [43, 44]. Thus, the ACSL4/LPCAT3 signalling pathway can activate lipid peroxidation to produce phospholipid hydroperoxides (PLOOHs) from PUFAs, thereby promoting ferroptosis [20, 21, 45]. ACSL4 can remodel the lipid composition of cells, especially AA and Ada, which in turn determines their sensitivity to ferroptosis [44]. We found that *TCP1* knockdown in GCB DLBCL downregulated ACSL4 expression, resulting in ferroptosis resistance, while also downregulating the ACSL4/LPCAT3 signalling pathway. Moreover, ACSL4 is degraded through the ubiquitin/proteasome pathway [46]. In our study, compared with control cells, *TCP1*-knockdown DB cells exhibited higher levels of ACSL4 ubiquitination, promoting its degradation. This may underlie the positive regulation of ferroptosis by *TCP1* in GCB DLBCL cells.

High TCP1 expression is associated with poor prognosis in various tumours. By measuring TCP1 expression levels in tumour tissues, we found higher TCP expression levels in patients with DLBCL than in lymph node tissues with reactive hyperplasia, which was associated with significantly shorter OS. Univariate and multivariate analyses demonstrated that high TCP1 expression was an independent prognostic factor for DLBCL. This was verified in the GEO dataset GSE10846 but not supported by GSE53786 and GEPIA data. Owing to the prevalent differences in prognosis between the GCB and non-GCB subtypes, our subgroup analysis revealed that although there was no difference in TCP1 positivity between the GCB and non-GCB groups, TCP1 positivity was associated with poor prognosis in patients with non-GCB but did not contribute to poor prognosis in those with GCB. Therefore, TCP1 may fulfil different functions in different subtypes of DLBCL cells.

This study has several limitations. We did not examine the mechanisms that regulate TCP1 in different DLBCL subtypes, leading to its different functions, or the mechanisms by which TCP1 leads to poor prognosis in the non-GCB subtype. We are currently conducting further comprehensive investigations to explore these issues.

In conclusion, our findings suggest that the molecular heterogeneity of TCP1 expression can be used to discriminate between DLBCL subtypes regarding their prognosis; high TCP1 expression predicts poor prognosis in the non-GCB subtype. In the GCB subtype, TCP1 stabilises ACSL4 and reduces its ubiquitination and degradation, thereby activating the ACSL4/LPCAT3 signalling pathway, altering the cellular lipid composition, and promoting RSL3-induced ferroptosis. These results may benefit the precision treatment of patients with different DLBCL subtypes.

### Supplementary information

supplementary Table 1

supplementary Table 2

western blot

## Data Availability

The datasets used and/or analysed during the current study are available from the corresponding author upon reasonable request.
