# Supplementary material for: TCP1 expression alters the ferroptosis sensitivity of diffuse large B-cell lymphoma subtypes by stabilising ACSL4 and influences patient prognosis
Source: Cell Death Dis. 2024 Aug 22;15(8):611. doi: 10.1038/s41419-024-07001-0 (PMC11341815; doi:10.1038/s41419-024-07001-0)

West blot

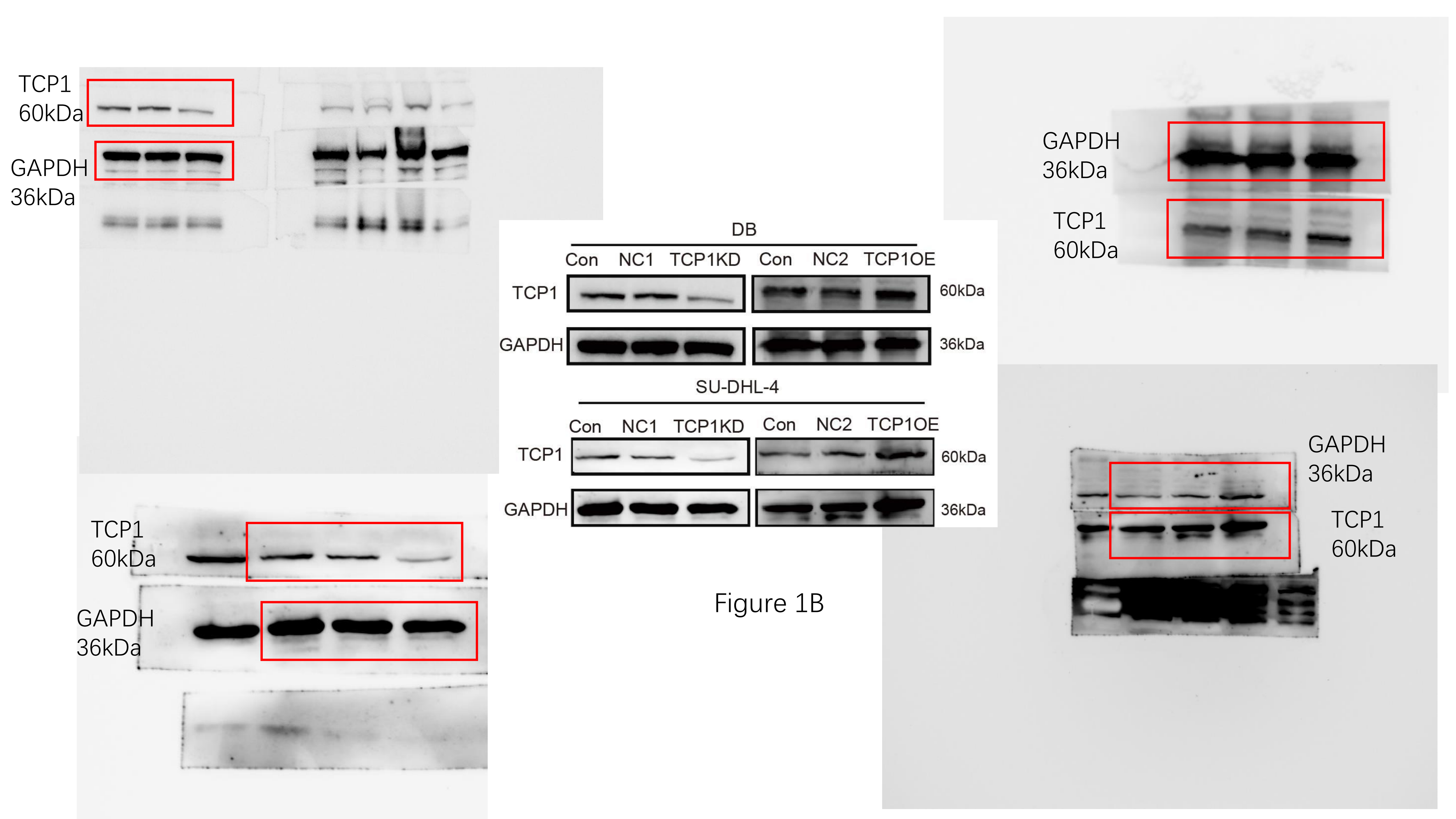
TCP1
60kDa
GAPDH
36kDa
DB
Con
NC1
TCP1KD
Con
NC2
TCP1OE
TCP1
60kDa
GAPDH
36kDa
SU-DHL-4
Con
NC1
TCP1KD
Con
NC2
TCP1OE
TCP1
60kDa
GAPDH
36kDa
GAPDH
36kDa
TCP1
60kDa
TCP1
60kDa
GAPDH
36kDa
GAPDH
36kDa
TCP1
60kDa

Figure 1B

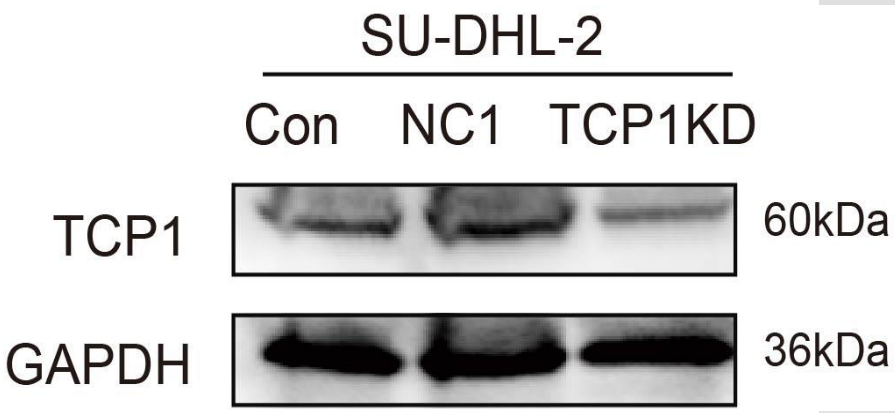
SU-DHL-2
Con
NC1
TCP1KD
TCP1
60kDa
GAPDH
36kDa

Figure 1E

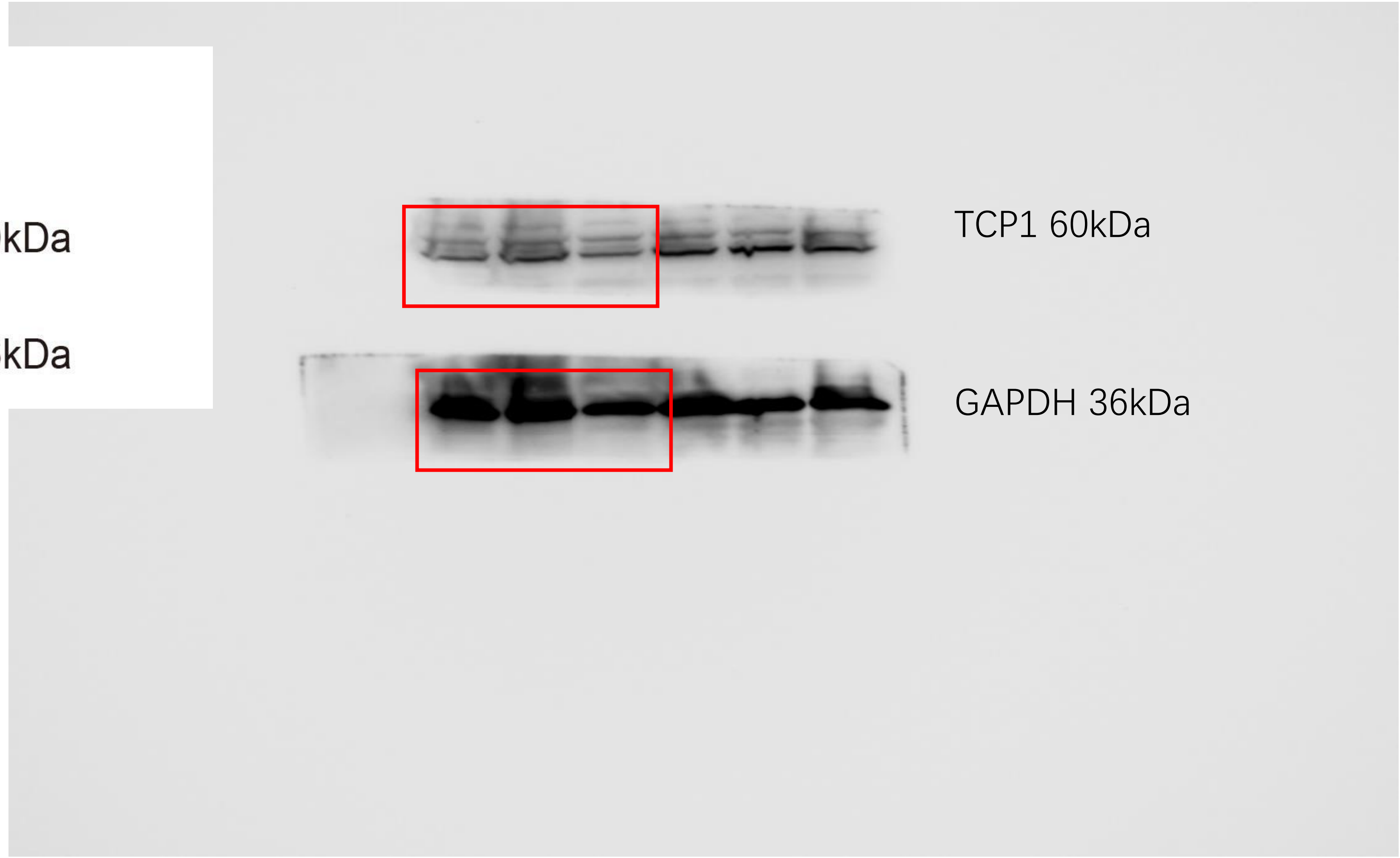
TCP1 60kDa
GAPDH 36kDa

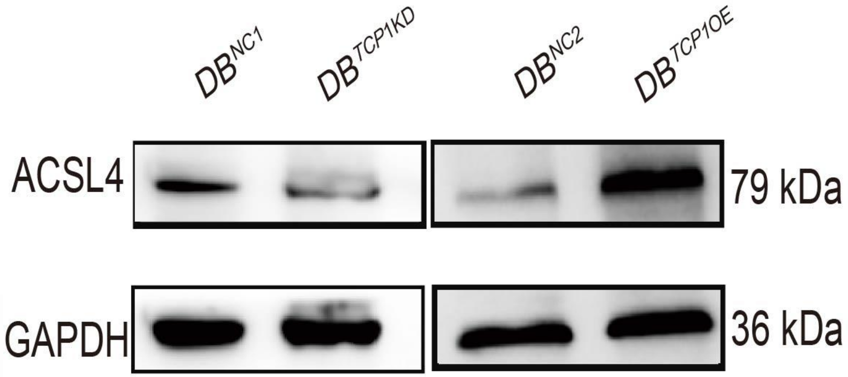
DB^NC1
DB^TCP1KD
DB^NC2
DB^TCP1OE
ACSL4
79 kDa
GAPDH
36 kDa

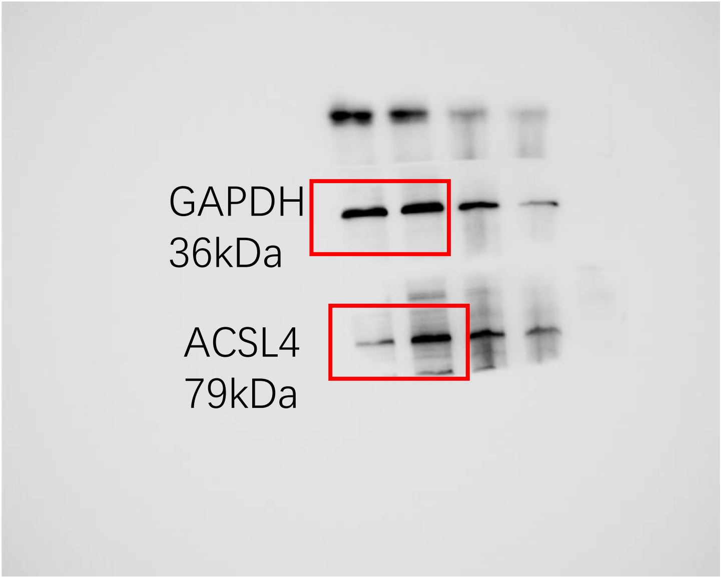
GAPDH
36kDa
ACSL4
79kDa

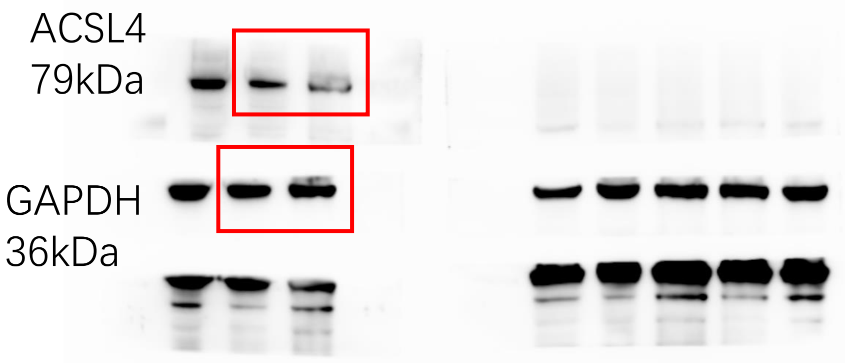
ACSL4
79kDa
GAPDH
36kDa

Figure 3C

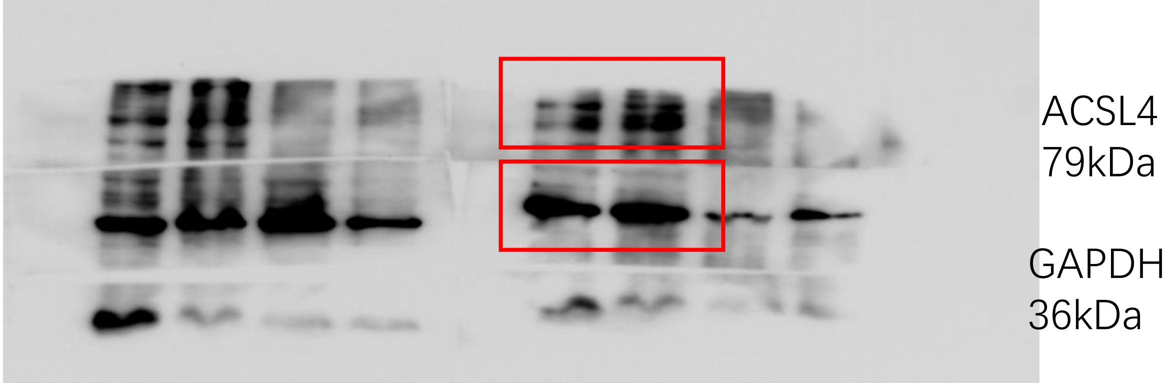
ACSL4
79kDa
GAPDH
36kDa

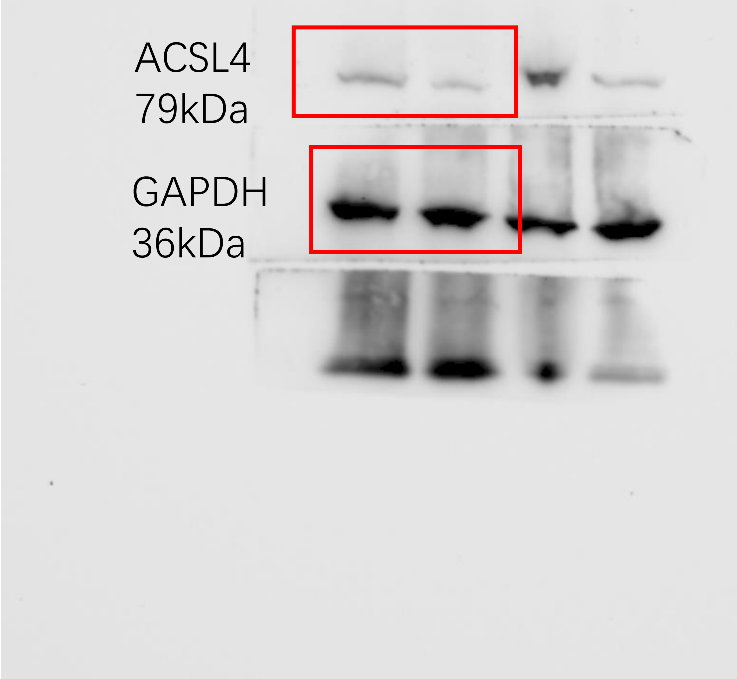
ACSL4
79kDa
GAPDH
36kDa

D

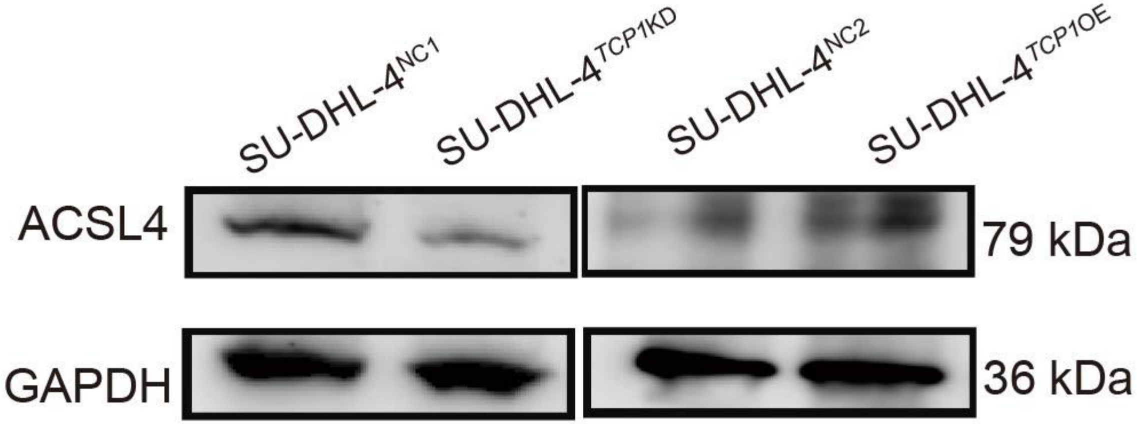
SU-DHL-4NC1
SU-DHL-4TCP1KD
SU-DHL-4NC2
SU-DHL-4TCP1OE
ACSL4
GAPDH
79 kDa
36 kDa

Figure 3D

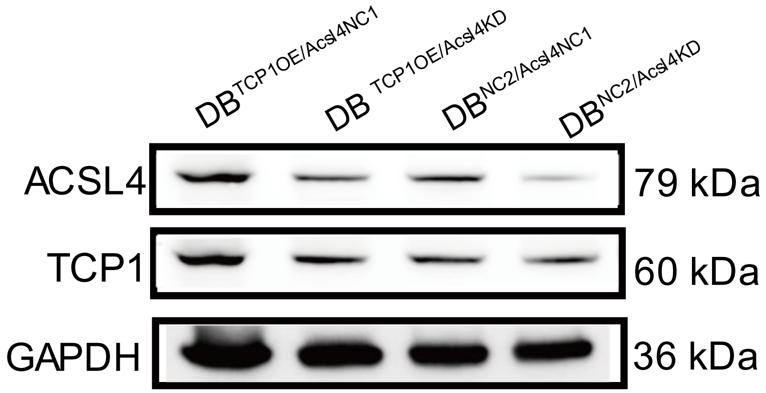

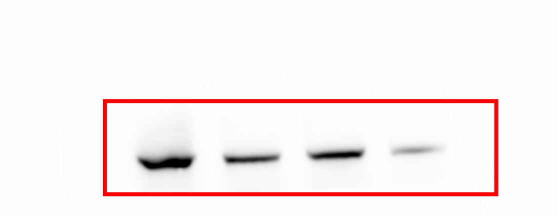

ACSL4
79kDa

Figure 3G

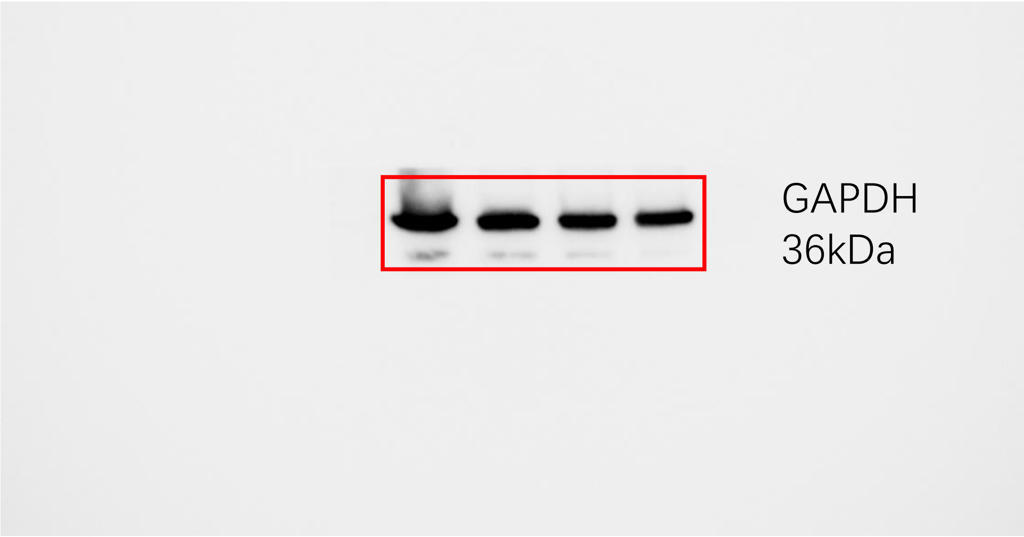

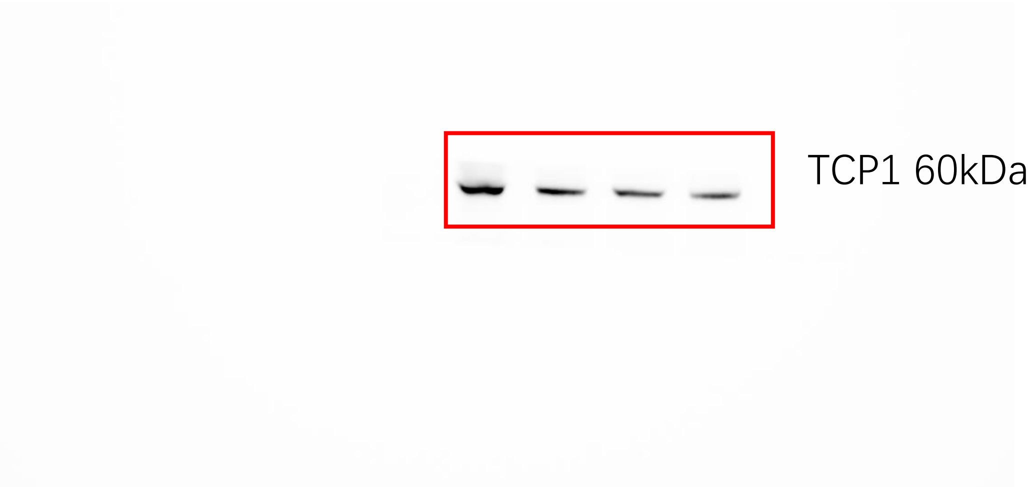

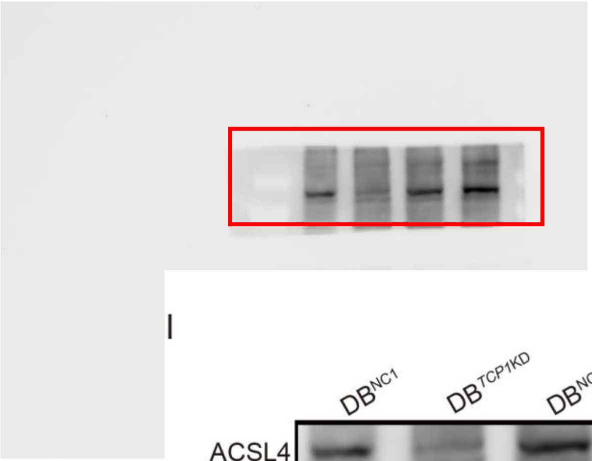
ACSL4
79kDa

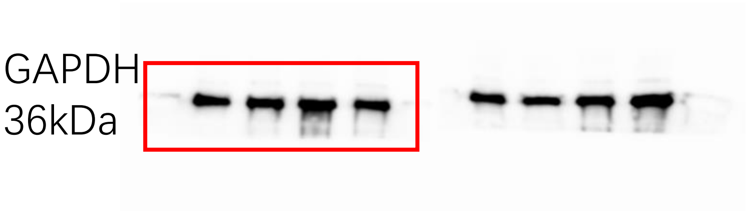
GAPDH
36kDa

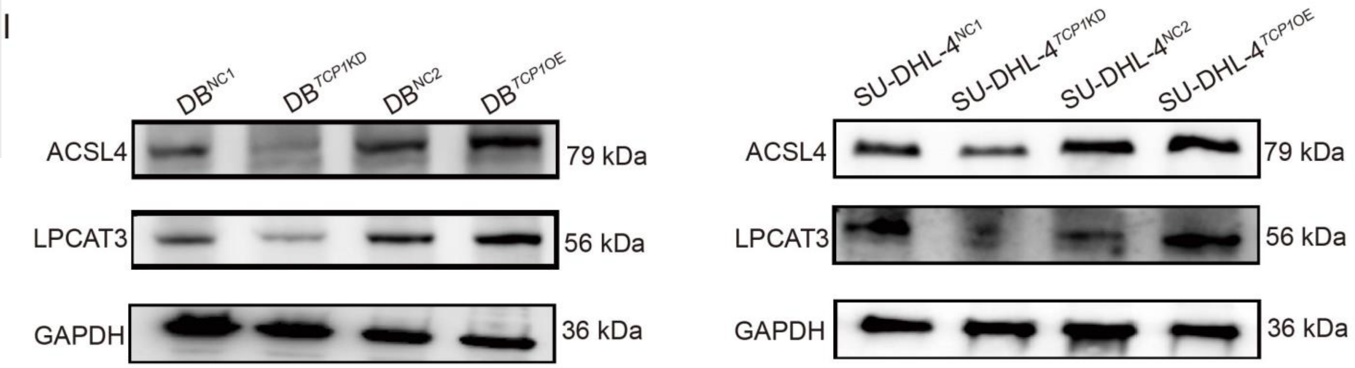
I
DB^NC1
DB^TCP1KD
DB^NC2
DB^TCP1OE
ACSL4
79 kDa
LPCAT3
56 kDa
GAPDH
36 kDa
SU-DHL-4^NC1
SU-DHL-4^TCP1KD
SU-DHL-4^NC2
SU-DHL-4^TCP1OE
ACSL4
79 kDa
LPCAT3
56 kDa
GAPDH
36 kDa

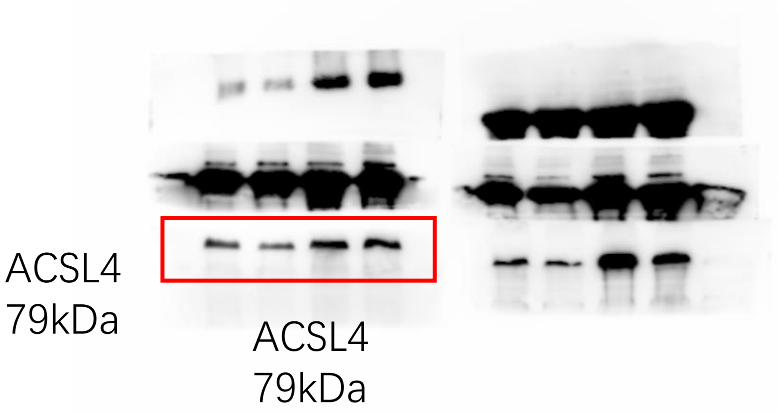
ACSL4
79kDa
ACSL4
79kDa

Figure 3I

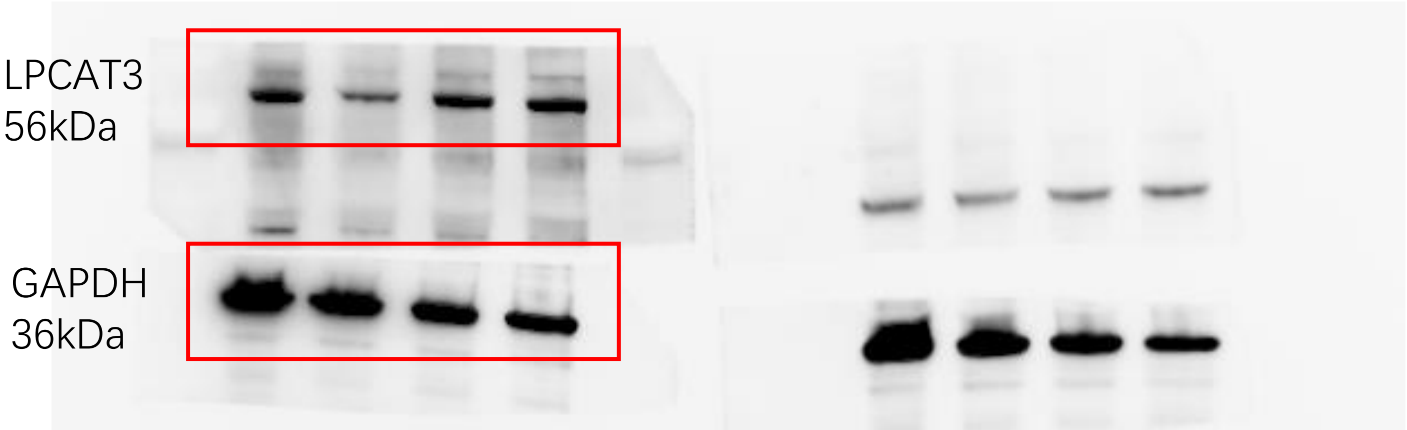
LPCAT3
56kDa
GAPDH
36kDa

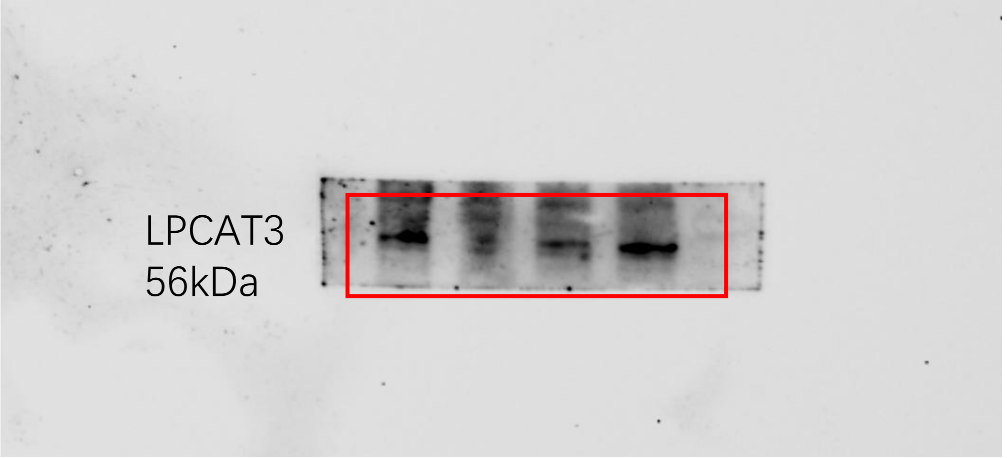
LPCAT3
56kDa

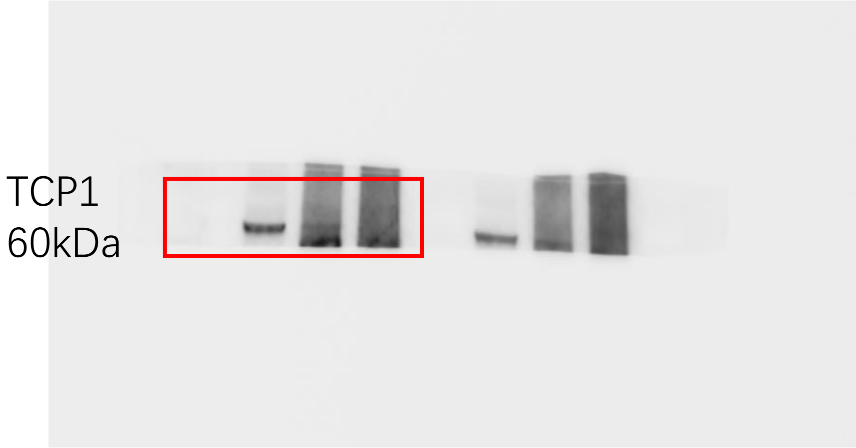
TCP1
60kDa

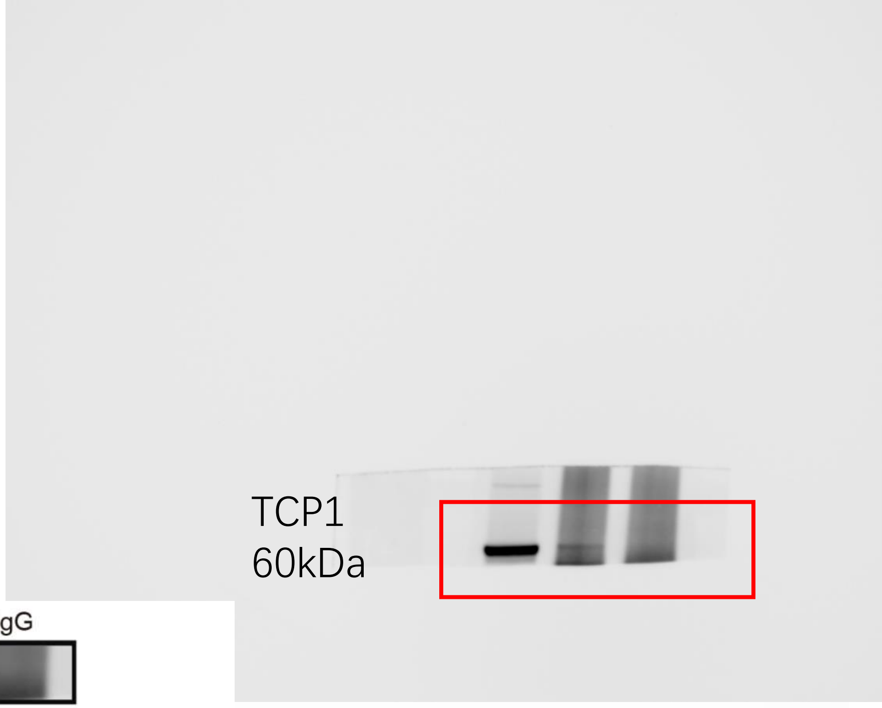
TCP1
60kDa

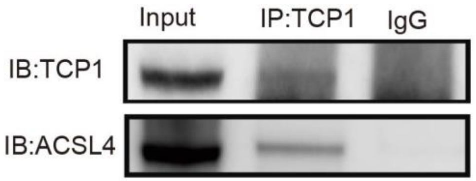
Input
IP:TCP1
IgG
IB:TCP1
IB:ACSL4

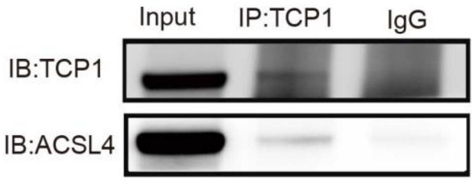
Input
IP:TCP1
IgG
IB:TCP1
IB:ACSL4

Figure 4B

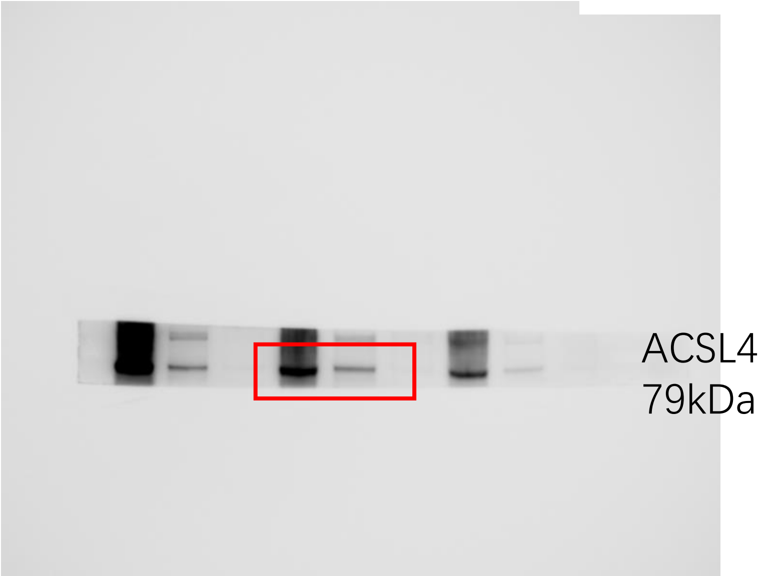
ACSL4
79kDa

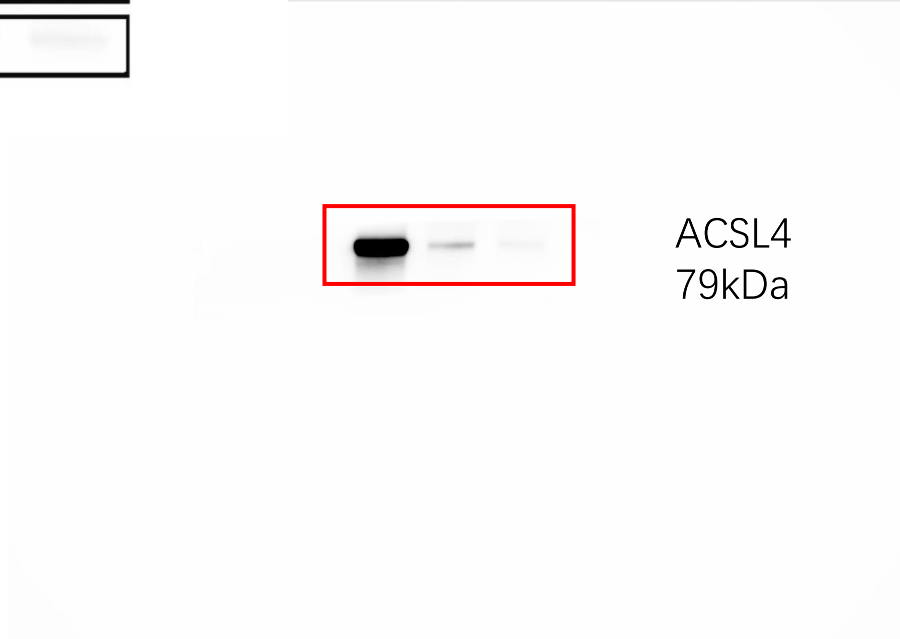
ACSL4
79kDa

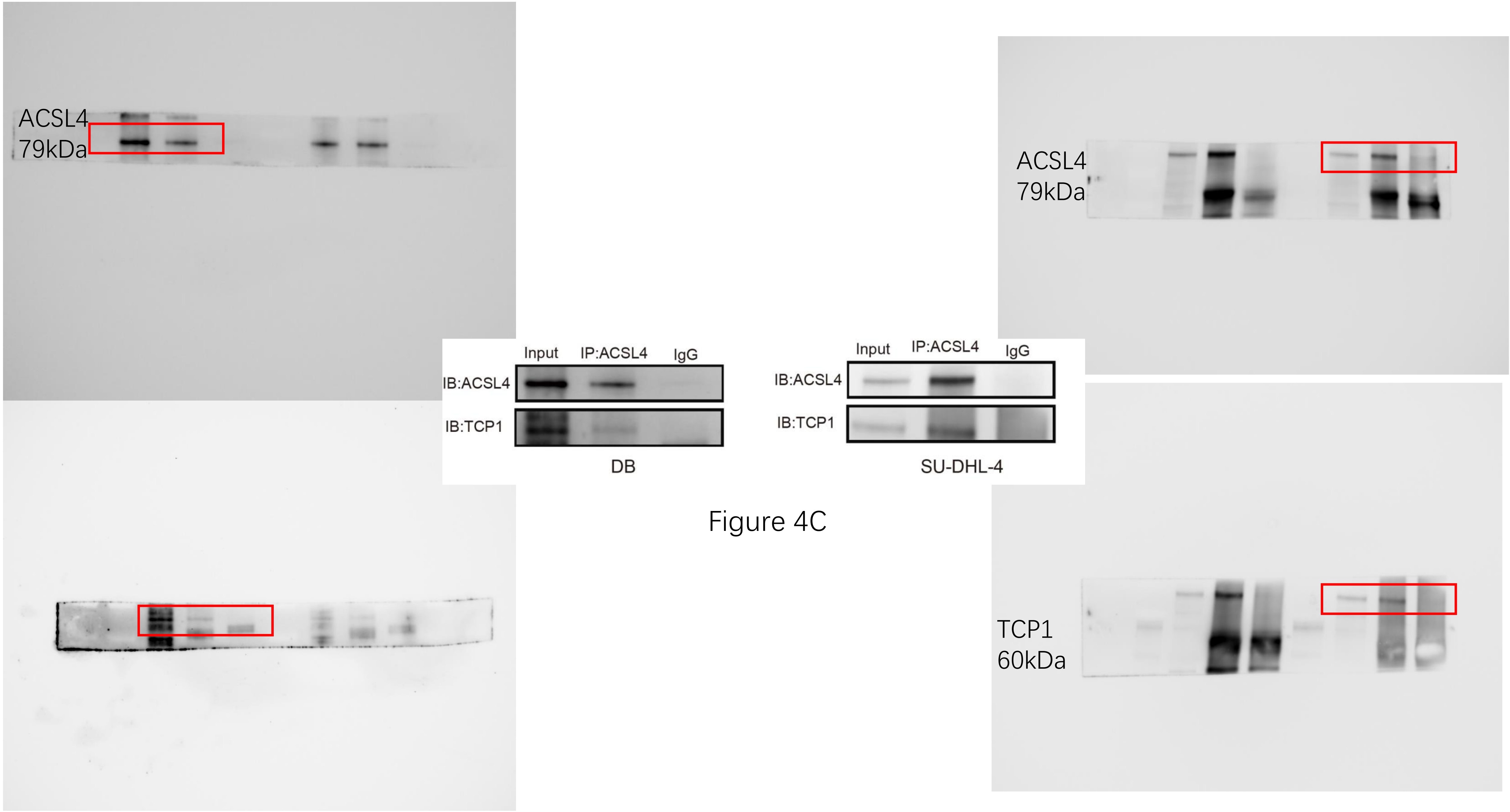
ACSL4
79kDa
Input
IP:ACSL4
IgG
IB:ACSL4
IB:TCP1
DB
Input
IP:ACSL4
IgG
IB:ACSL4
IB:TCP1
SU-DHL-4
ACSL4
79kDa
TCP1
60kDa

Figure 4C

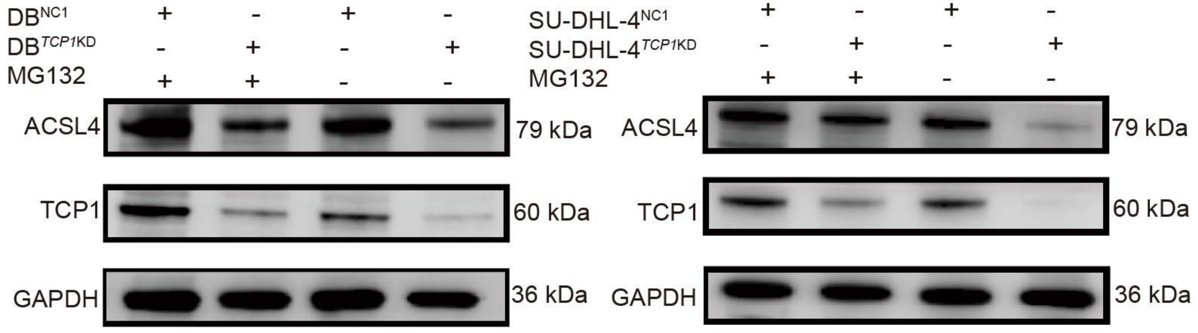
DB^NC1 + - + -
DB^TCP1KD - + - +
MG132 + + - -
ACSL4
79 kDa
TCP1
60 kDa
GAPDH
36 kDa
SU-DHL-4^NC1 + - + -
SU-DHL-4^TCP1KD - + - +
MG132 + + - -
ACSL4
79 kDa
TCP1
60 kDa
GAPDH
36 kDa

Figure 4D

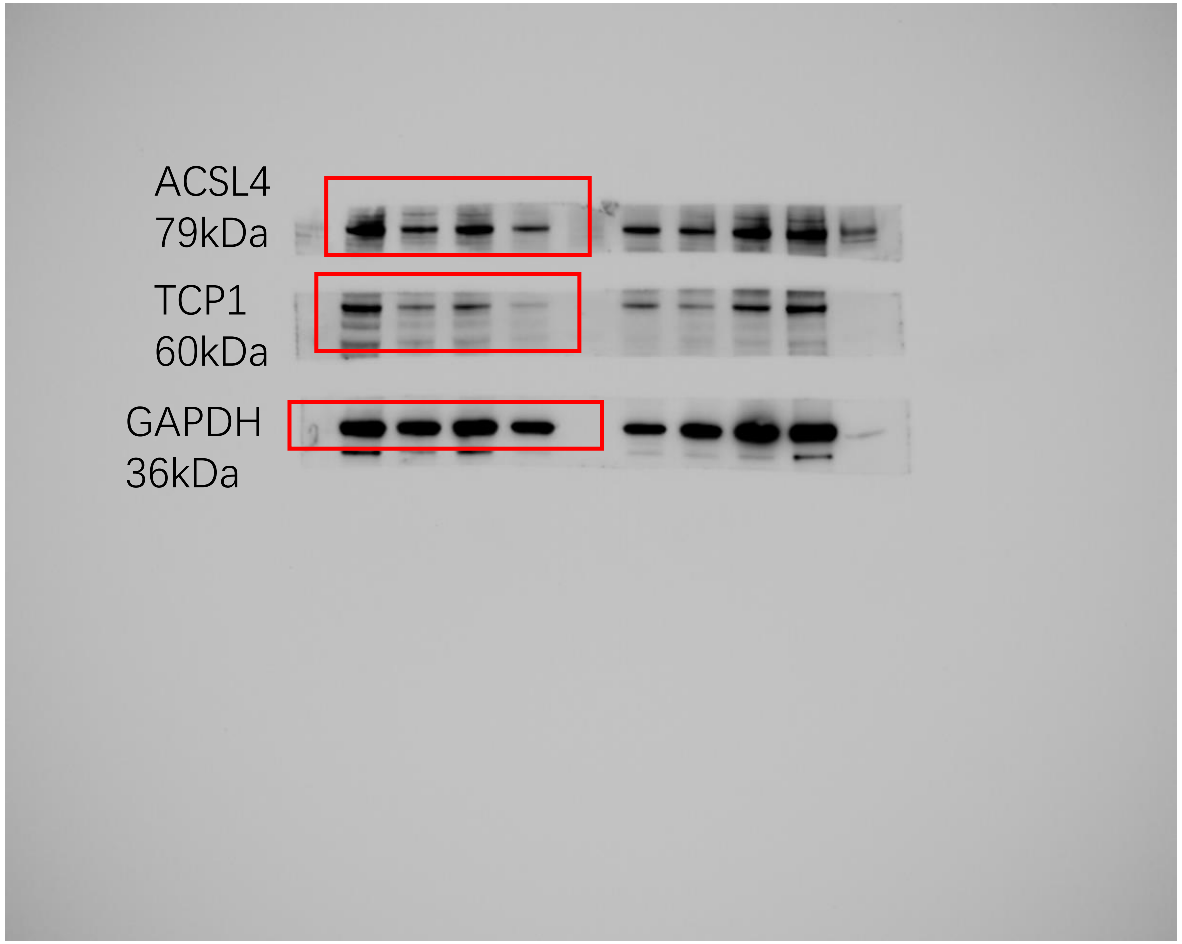
ACSL4
79kDa
TCP1
60kDa
GAPDH
36kDa

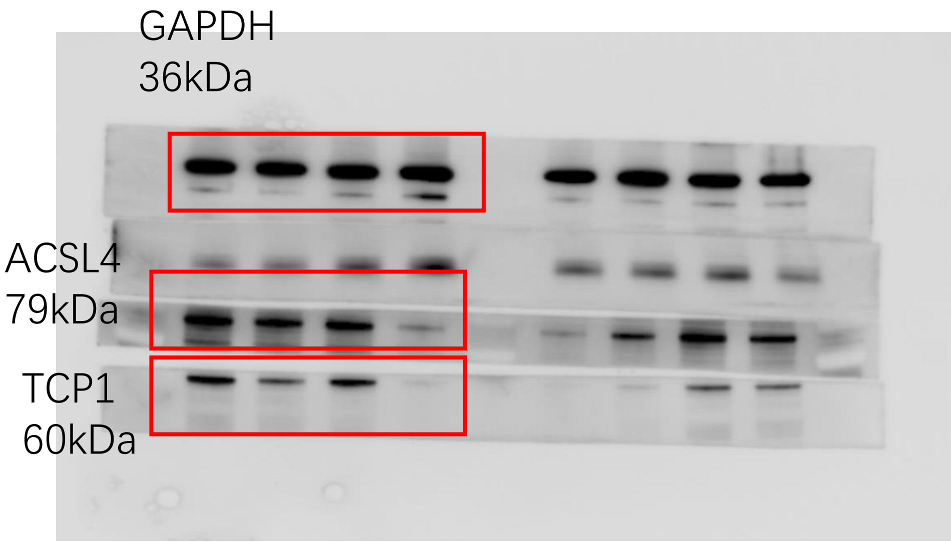
GAPDH
36kDa
ACSL4
79kDa
TCP1
60kDa

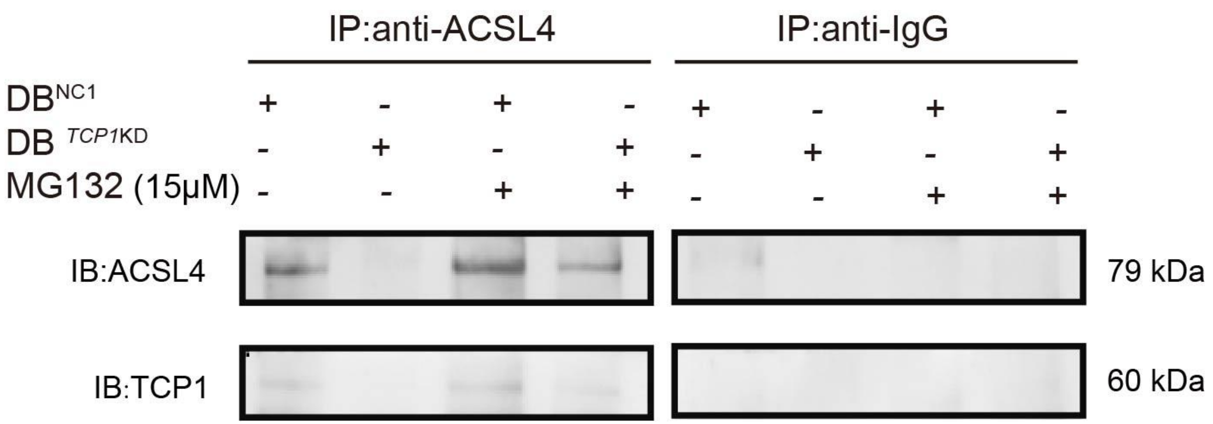
IP:anti-ACSL4
IP:anti-IgG
DBNC1 + - + - + - + -
DB TCP1KD - + - + - + - +
MG132 (15μM) - - + + - - + +
IB:ACSL4
79 kDa
IB:TCP1
60 kDa

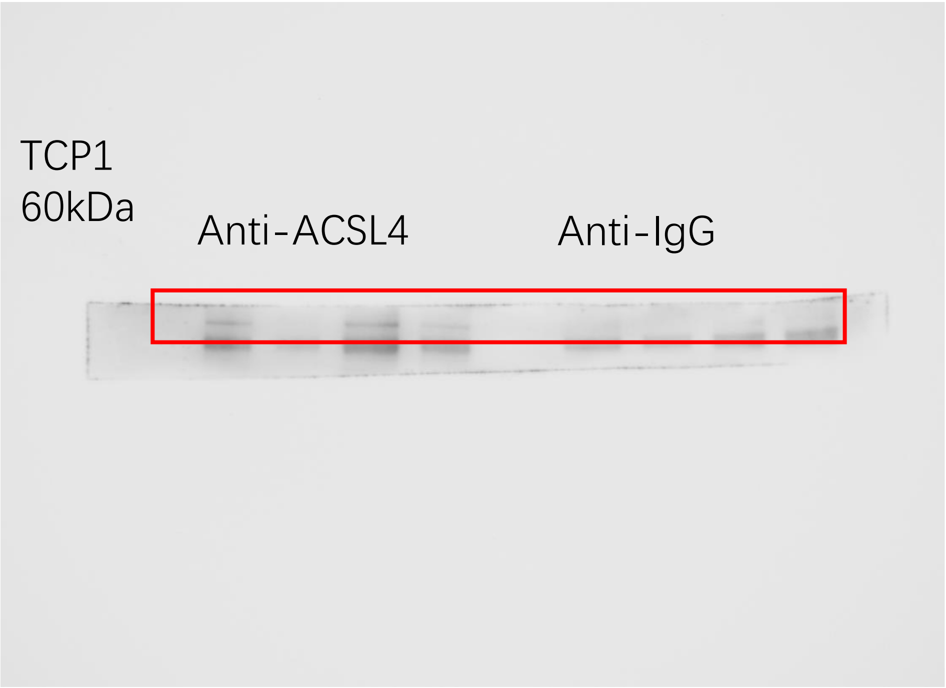
TCP1
60kDa
Anti-ACSL4
Anti-IgG

Figure 4E

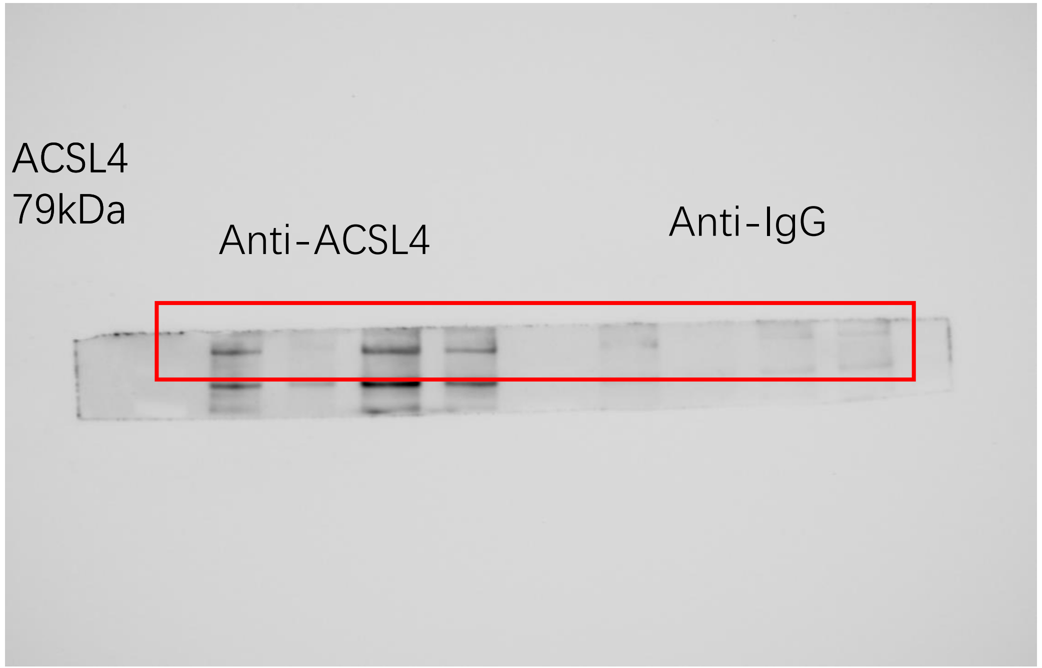
ACSL4
79kDa
Anti-ACSL4
Anti-IgG

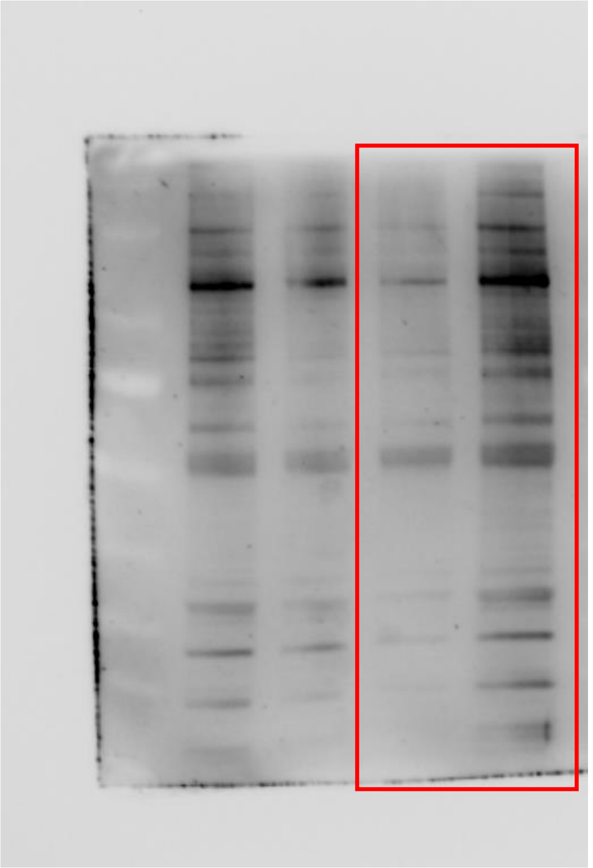

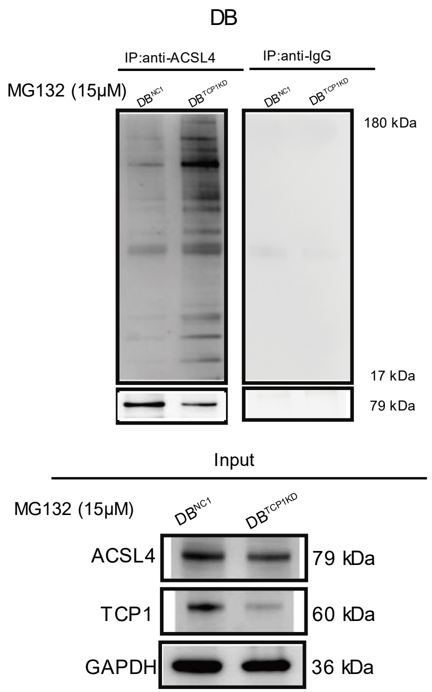

DB
IP:anti-ACSL4
IP:anti-IgG
MG132 (15μM)
DB^NC1
DB^TCP1KD
DB^NC1
DB^TCP1KD
180 kDa
17 kDa
79 kDa
Input
MG132 (15μM)
DB^NC1
DB^TCP1KD
ACSL4
79 kDa
TCP1
60 kDa
GAPDH
36 kDa

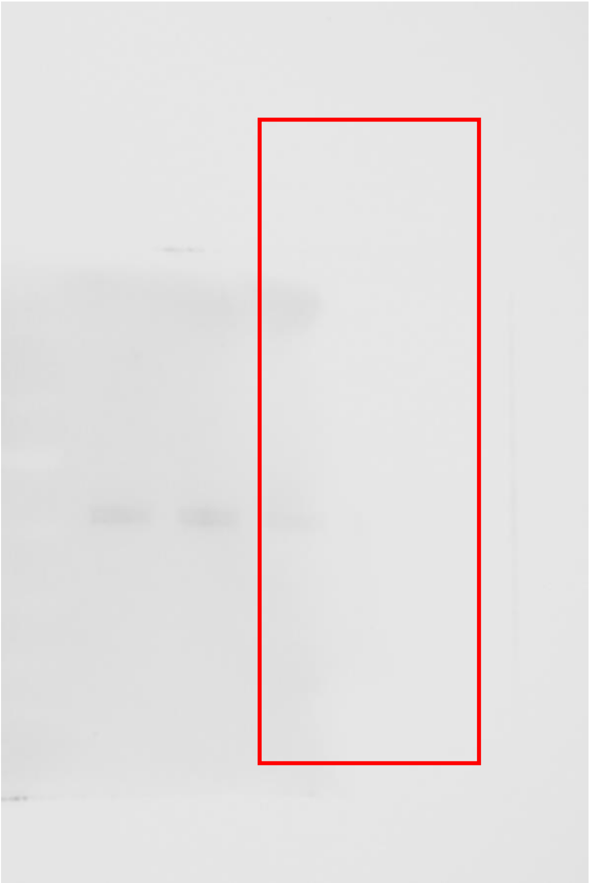

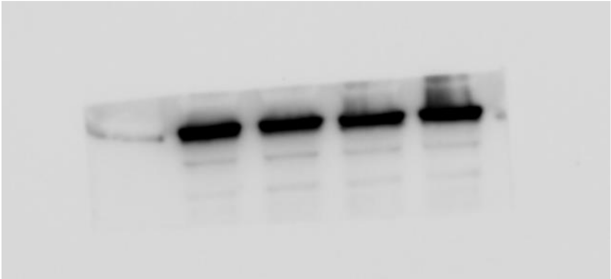

Figure 4F

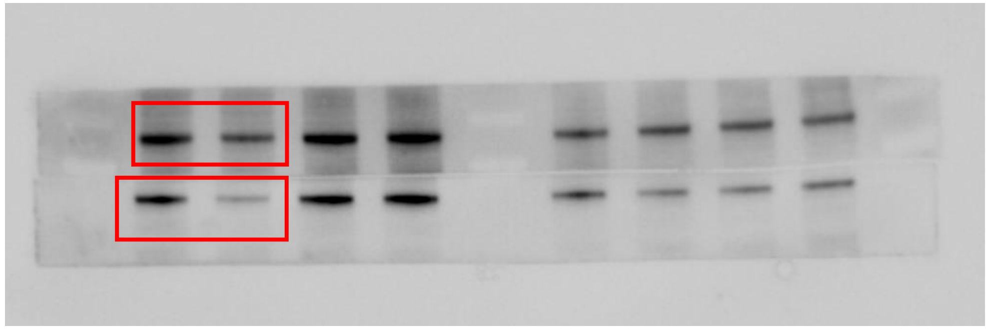

Supplement: Supplementary file 3 — western blot [file 41419_2024_7001_MOESM3_ESM.pdf]
